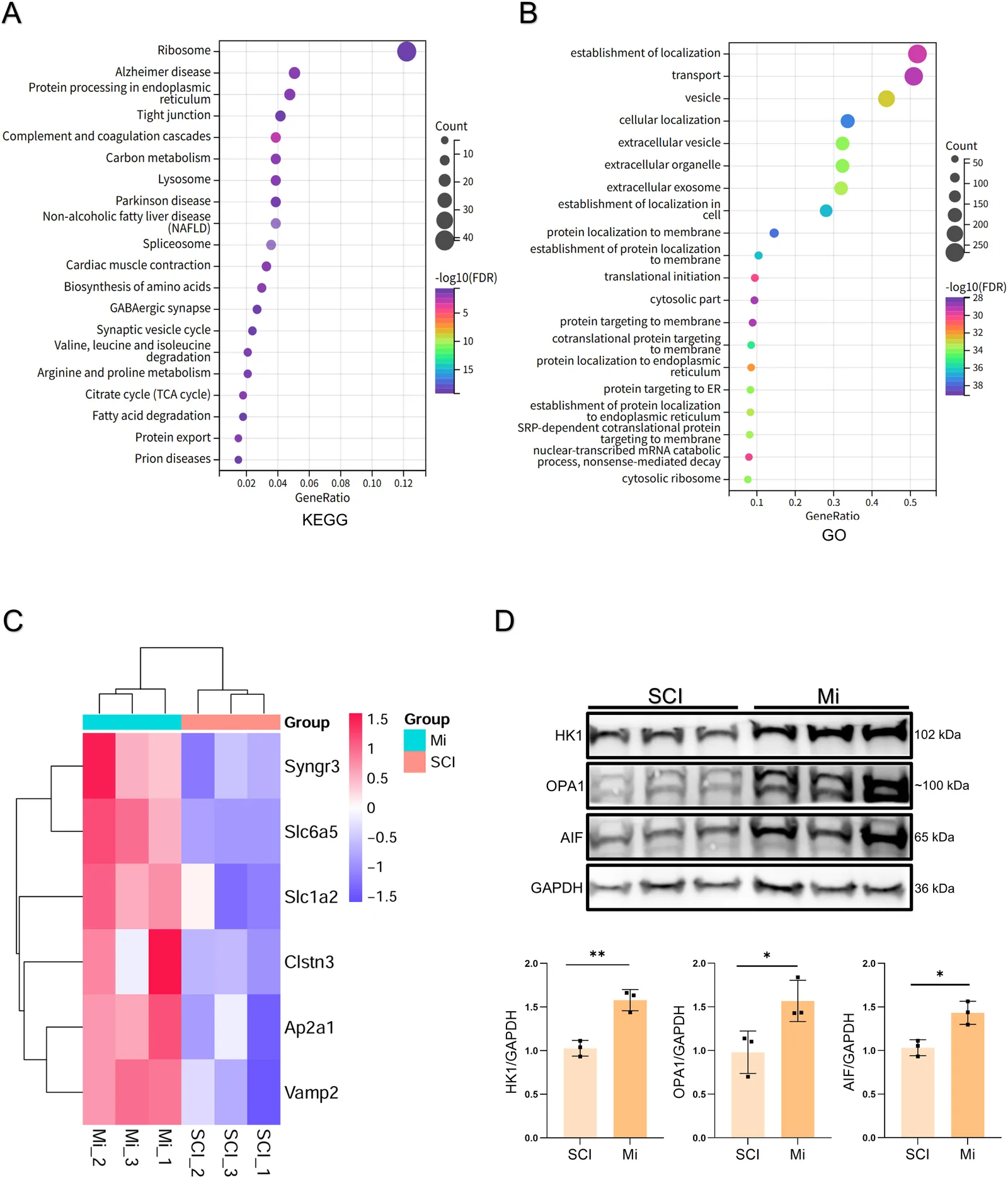# Transplantation of LPS, IL-4, and TGF-β-induced reparative-biased macrophages promotes early motor functional recovery after spinal cord injury in rats

**DOI:** 10.1016/j.jot.2026.101219

**Published:** 2026-09-05

**Authors:** Xiaowei Zha, Guoli Zheng, Hao Wang, Obada T. Alhalabi, Raban Heller, Maryam Hatami, Thomas Skutella, Marcin Luzarowski, Xingjin Wang, Sandro M. Krieg, Andreas Unterberg, Alexander Younsi

**Affiliations:** a Department of Neurosurgery, Heidelberg University Hospital, Heidelberg, Germany; b Medical Faculty, University of Heidelberg, Heidelberg, Germany; c Institute for Experimental Endocrinology, Charité—Universitätsmedizin Berlin, corporate member of Freie Universität Berlin, Humboldt-Universität zu Berlin, Berlin Institute of Health, Berlin, Germany; d Department of Food Chemistry and Toxicology, Technische Universität Berlin, Berlin, Germany; e Department of Trauma Surgery and Orthopaedics, Reconstructive and Septic Surgery, Sports Traumatology, German Armed Forces Hospital, Ulm, Germany; f Department of Neuroanatomy, University of Heidelberg, Heidelberg, Germany; g Core Facility for Mass Spectrometry and Proteomics, Center for Molecular Biology of Heidelberg University (ZMBH), DKFZ-ZMBH Alliance, Heidelberg, Germany; h Department of Neurosurgery, BG Klinik Ludwigshafen, Ludwigshafen, Germany

**Keywords:** Cell transplantation, Macrophages, Neuroinflammation, Regeneration, Spinal cord injury, Tissue repair

## Abstract

**Objective:**

Spinal cord injury (SCI) triggers an inflammatory cascade that often culminates in chronic neurological deficits. This study investigates a combined stimulation approach using lipopolysaccharide (LPS), interleukin-4 (IL-4), and transforming growth factor-beta (TGF-β) to generate reparative-biased macrophages and evaluate their early effects after SCI.

**Methods:**

Bone marrow-derived macrophages (BMDMs) from Wistar rats were exposed to LPS, IL-4, and TGF-β for 24 h to generate reparative-biased hybrid macrophages (Mi) in vitro. The anti-inflammatory and pro-regenerative markers of Mi were characterised by qPCR, immunocytochemistry, and proteomic analysis via mass spectrometry. *In vivo*, rats with a T9/10 clip-contusion-compression SCI received either Mi transplantation or direct inducer administration at the lesion site. Histological outcomes and functional recovery were assessed and compared between injured and uninjured controls over a 14-day period.

**Results:**

Following 24 h induction, Mi exhibited significantly elevated mRNA and immunofluorescence expression of Arg1, as well as increased IL-10 mRNA levels, compared to naïve BMDMs in vitro, while iNOS expression remained unchanged. Bioinformatic analysis of proteomic data from Mi revealed enrichment in metabolic activity, immune modulation, and phagocytosis pathways. Fluorescent microsphere phagocytosis assays demonstrated that Mi macrophages retained robust phagocytic activity. *In vivo*, transplantation of Mi resulted in elevated Arg1 expression and reduced iNOS and IL-1β levels 3 days after SCI, suggesting an anti-inflammatory shift. Immunohistochemical analysis demonstrated significantly reduced lesions and demyelinated areas, as well as increased preservation of motor neurons and oligodendrocytes. Mi transplantation was also associated with reduced apoptosis and improved early functional recovery at 14 days after SCI.

**Conclusion:**

This study suggests that LPS, IL-4, and TGF-β-induced Mi macrophages display a reparative-biased hybrid profile that modulates inflammation, supports early tissue preservation, and promotes functional recovery after SCI, supporting Mi transplantation as a potential cell-based therapeutic strategy for further investigation.

**The translational potential of this article:**

Transplantation of this reparative-biased hybrid macrophage phenotype may provide a potential therapeutic strategy for the treatment of spinal cord injury.

## Graphical abstract

**Figure:**
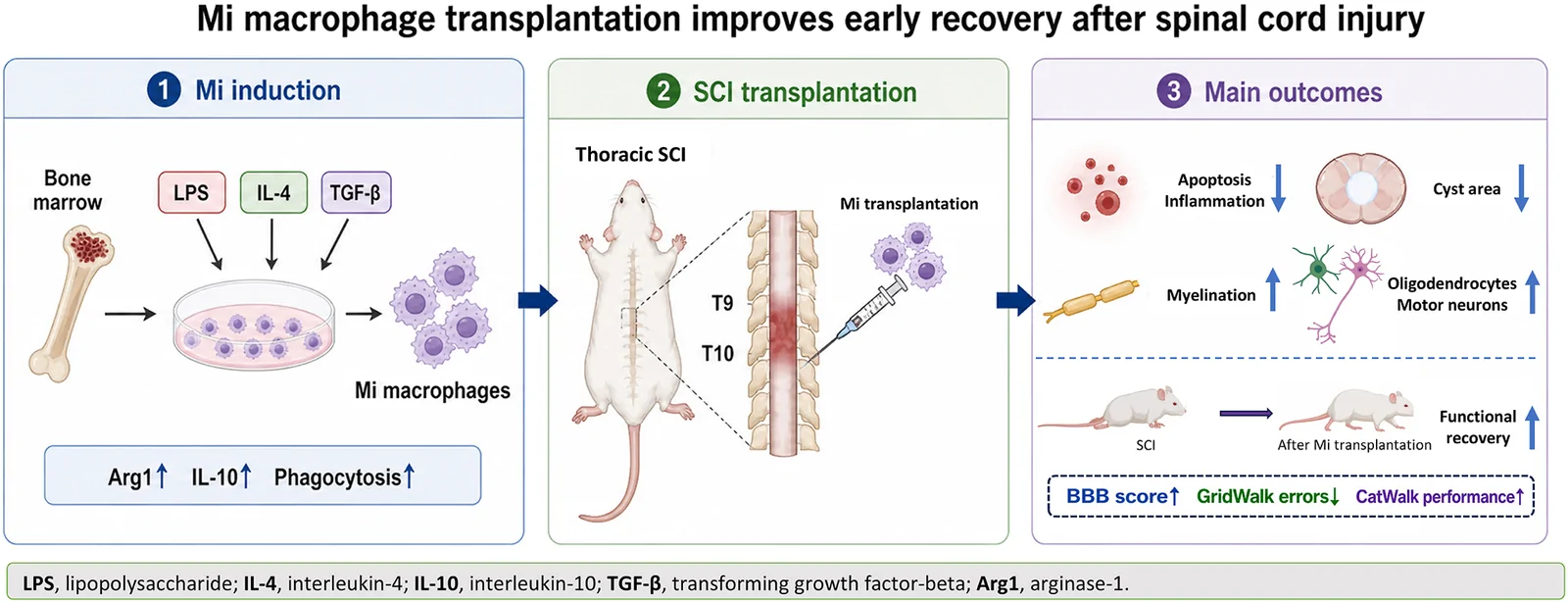

## 1 Introduction

Spinal cord injury (SCI) is a severe neurological dysfunction typically caused by traumatic events such as motor vehicle accidents, sports injuries, or falls. However, nontraumatic causes like degenerative disc disease, infections, or tumours also exist [1]. Beyond the profound physical and emotional impact on affected individuals, SCI imposes a substantial economic burden on families and healthcare systems worldwide [2]. It is estimated that over two million people globally are living with the consequences of SCI, underscoring the urgent need for effective therapies to restore function and quality of life [3].

The pathophysiology of SCI involves two distinct but interconnected phases. The primary injury phase is characterised by direct mechanical damage to the spinal cord, resulting from mechanisms such as laceration, compression, transection, shearing, or stretching, which immediately disrupts neural pathways. The secondary injury phase, which evolves over days to months, encompasses a complex cascade of events, including ischaemic injury, excitotoxicity, cell death, and a robust immune-inflammatory response that can further exacerbate tissue damage [4,5]. In particular, inflammation plays a pivotal role in modulating the extent of secondary injury and the potential for neural regeneration [6,7].

Macrophages are central players in this inflammatory response. Following SCI, macrophages and microglia rapidly accumulate at the lesion site, adopting either a pro-inflammatory M1 phenotype, which secretes cytokines and phagocytoses debris, or an anti-inflammatory M2 phenotype, which supports tissue repair and regenerative processes [2]. While M1 macrophages contribute to debris clearance and initial defence, prolonged M1 activation is associated with exacerbated tissue damage. Conversely, M2 macrophages promote resolution of inflammation and tissue healing [8]. Recent studies have highlighted the critical roles of both phenotypes in different phases of SCI recovery [9]. However, the traditional M1/M2 paradigm is now recognized as an oversimplification, as macrophage activation is a dynamic and plastic process, with intermediate and hybrid states emerging in response to complex injury signals [10].

Building on the evolving understanding of macrophage plasticity, this study sought to generate reparative-biased macrophages by integrating pro- and anti-inflammatory activation cues through simultaneous exposure of bone marrow–derived macrophages to lipopolysaccharide (LPS), interleukin-4 (IL-4), and transforming growth factor-β (TGF-β). This combined stimulation was used to generate a reparative-biased macrophage population, designated Mi, which was characterised in vitro and evaluated in vivo after transplantation into the injured spinal cord of a rat SCI model. We hypothesised that Mi macrophages would acquire a reparative-biased hybrid profile and promote early tissue preservation and functional recovery after SCI by modulating the inflammatory microenvironment.

## 2 Materials and methods

### 2.1 Isolation, culture, and induction of bone marrow-derived macrophages (BMDMs)

Bone marrow cells were flushed from rat femurs with cold DPBS, pelleted, and subjected to ACK-mediated erythrocyte lysis (Lonza, BP10-548E, Switzerland). Cells were resuspended in complete Dulbecco’ modified Eagle's medium (DMEM, 11965092, Gibco, New York, USA) containing 10% fetal bovine serum (FBS, A5256701, Thermo Fisher, Massachusetts, USA) and 1% penicillin–streptomycin (PS, 15140122, Sigma–Aldrich, Missouri, USA), and pre-adhered for 4 h to remove resident macrophages. Non-adherent cells were then cultured in complete DMEM supplemented with 25 ng/mL rat macrophage colony-stimulating factor (rM-CSF, 556902, BioLegend, CA, USA) at 2 × 10^6^ cells/mL. After 3 days, half of the medium was replaced with fresh rM-CSF–containing medium, and cells were cultured for an additional 2 days to generate BMDMs. Finally, the BMDMs were harvested and seeded for the following experiments (Fig. S1A and B).

For polarisation, BMDMs were stimulated for 24 h in complete DMEM containing 10% FBS and 1% penicillin–streptomycin, supplemented with IFN-γ (20 ng/mL; 570202, BioLegend, CA, USA) and LPS (100 ng/mL; L2630, Sigma, St. Louis, MO, USA) for M1 induction, IL-4 (20 ng/mL; 776902, BioLegend, CA, USA) and IL-13 (10 ng/mL; 751702, BioLegend, CA, USA) for M2 induction, or LPS (10 ng/mL), IL-4 (40 ng/mL), and TGF-β (20 ng/mL; 7754-BH, R&D Systems, Minneapolis, MN, USA) for Mi induction. The medium was not replaced during the 24 h induction period. The concentrations of all macrophage-inducing factors were selected based on previously reported macrophage stimulation conditions and preliminary dose-selection tests [[11], [12], [13], [14]]. After induction, cells were washed three times with sterile PBS to remove residual soluble inducers and subsequently harvested for RNA extraction and quantitative polymerase chain reaction (qPCR) or fixed with 4% paraformaldehyde (PFA) for immunocytochemistry (ICC) and imaging. All experiments were performed in five independent replicates. For transplantation, washed Mi macrophages were resuspended in sterile PBS and used only when viability exceeded 95% by trypan blue exclusion. To functionally assess residual biologically active inducers, an aliquot of washed Mi cells was briefly resuspended in complete DMEM; after cell removal by centrifugation, the resulting supernatant was applied to naïve BMDMs for 24 h before qPCR analysis of macrophage markers, with no detectable marker changes observed compared with fresh complete medium (Fig. S2A).

### 2.2 Primary neurons’ isolation, culture, and co-culture with macrophages

Primary neuron cultures were established as previously described [15]. Cortical tissues were isolated from Wistar rat embryos at E19, enzymatically dissociated with 0.05% trypsin-EDTA (25200056, Thermo Fisher, Massachusetts, USA) for 15 min, and filtered through a 100-μm mesh to obtain single-cell suspensions. Cells were plated at 3 × 10^4^ cells/well on poly-L-lysine-coated 24-well plates in complete DMEM, which was replaced after 4–6 h with neural culture medium (Neurobasal Plus (21103-049, Gibco, New York, USA) supplemented with 2% B-27 Plus (17504-044, Gibco, New York, USA), 1% GlutaMAX (35050061, Gibco, New York, USA), and 1% penicillin–streptomycin). Medium was changed after 24 h and partially renewed weekly thereafter. To co-culture with macrophages, the medium was changed to Neurobasal medium without the B-27 supplement, and BMDMs were overlaid (neuron:macrophage ratio of 4:1). Macrophages were induced by supplementing the co-culture medium with the indicated cytokine combinations. After 24 h, cells were fixed in 4% PFA and subjected to immunocytochemistry and imaging analyses. All experiments were performed in five independent replicates.

### 2.3 Immunocytochemistry staining

The fixed cells were initially made permeable by incubating them for 10 min with PBST (PBS with 0.1% Tween® 20, P1379, Sigma–Aldrich, Missouri, USA), including 0.3% Triton X-100 (X100, Sigma–Aldrich, Missouri, USA). A blocking solution comprised of 1% BSA and 0.3% Triton-X-100 diluted in 1×PBS was administered to the wells or plates, followed by 1 h of incubation at room temperature. The cells were then incubated overnight at 4 °C with one of the following primary antibodies: anti-iNOS (1:250; ab178945, Abcam), anti-Arginase 1 (Arg1) (1:200; ab91279, Abcam), anti-CD11b (1:500; ab1211, Abcam), or anti-MAP2 (1:6000; NB300-213, Novus Biologicals). Afterwards, the cells were incubated with anti-rabbit Alexa 488 (1:500; ab150061; Abcam), anti-mouse Alexa 568 (1:500; ab175700; Abcam) or anti-chicken Alexa 647 (1:500; A21449; Invitrogen) as secondary antibodies at room temperature for 1 h. After washing the remaining secondary antibody away, the nuclei were stained with 4′,6′-diamidino-2-phenylindole dihydrochloride (DAPI). Image taking and analysis for the ICC staining are described below.

### 2.4 Quantitative polymerase chain reaction

Around 5 × 10^6^ macrophages were collected for Ribonucleic Acid (RNA) extraction from the in *vitro* experiments and rinsed with PBS. For RNA extraction from the in vivo experiments, spinal cord tissue was isolated and homogenized from the rats 3 days after SCI (see below). To isolate the total RNA from the cells or tissue, the NucleoSpin® RNA Isolation Kit (Macherey–Nagel GmbH & Co., Düren, Germany) was employed. Conversion to complementary Deoxyribonucleic Acid (cDNA) was done with the SuperScript II Reverse Transcriptase system (Invitrogen, Massachusetts, USA). The qPCR was performed using the StepOne Plus instrument (Applied Biosystems, Massachusetts, USA) according to the universal cycle conditions. The following primers were used: Arg1, forward primer, 5-GATGTGCCTCTGTCTTTTAGGG-3 and reverse primer, 5-AATGCTGCGGGACCTTTCTC-3; iNOS, forward primer, 5-CAGATCGAGCCCTGGAAGAC-3 and reverse primer, 5-TCCGCATTAGCACAGAAGCA-3; IL10, forward primer, 5-AAGAAGGACCAGCTGGACAAC-3 and reverse primer, 5-GCCTGGGGCATCACTTCTAC-3; IL-1β, forward primer, 5-AGGCTGACAGACCCCAAAAG-3 and reverse primer, 5-CTCCACGGGCAAGACATAGG-3; IL12, forward primer, 5-CATGGCTGGTGCACAGAAAC-3 and reverse primer, 5-ATGCTCGTCCACATGTCACC-3; TNF-α, forward primer, 5-AACACACGAGACGCTGAAGT-3 and reverse primer, 5-ATGCTCGTCCACATGTCACC-3; Actb, forward primer, 5-ATACCCACCATCACACCCTGG-3 and reverse primer, 5-AACCTTCTTGCAGCTCCTCCG-3.

The cycling thresholds (CTs) duplicates were averaged, and the relative amount of messenger RNA (mRNA) was computed using the 2^−ΔΔCT^ strategy [16]. Target gene relative expression levels were obtained by standardizing the data to rActb, a housekeeping gene. Five duplicate experiments were conducted for each.

### 2.5 Phagocytosis assay

Phagocytosis was assessed using an IgG–FITC phagocytosis assay kit (IgG FITC complex, Cayman Chemicals, Cat#Cay500290). FITC-labelled latex beads were added to macrophage cultures (1:200 dilution) and incubated for 2 h at 37 °C. Cells were washed with PBS, counterstained with Hoechst (Cayman Chemicals, Cat#Cay15547), and imaged using a Zeiss LSM700 confocal microscope (LSM 700, Carl Zeiss Microscopy GmbH, Jena, Germany). Five random fields per well were analysed, and mean fluorescence intensity (MFI) was quantified. All experiments were performed in five independent replicates.

### 2.6 Proteomic analysis

Proteomic analyses were performed on Mi and resting macrophages as well as spinal cord tissues from four experimental groups (n = 3 per group). Proteins were extracted in urea-based lysis buffer supplemented with protease and phosphatase inhibitors (78425, EDTA-free, ThermoFisher; 78420, ThermoFisher), quantified by Bradford assay, and digested using the SP3 method with minor modifications [17]. Peptides were desalted on C18 STAGE tips and analysed using an Ultimate 3000 liquid chromatography system coupled to an Orbitrap QE HF as described earlier [18]^,^ [19]. Raw data were searched against the Swiss-Prot *R. norvegicus* database using Proteome Discoverer, and downstream data processing and statistical analyses were performed in R and Rstudio (version 4.4.1) following established workflows [20]. Differentially expressed proteins (DEPs) between the sample groups were visualised. Analysis tools, including Gene Ontology (GO; https://geneontology.org/) and Kyoto Encyclopedia of Genes and Genomes (KEGG; https://www.kegg.jp/), Gene Set Enrichment Analysis (GSEA, https://www.gsea-msigdb.org/gsea/index.jsp), were utilised to investigate the biological functions of the DEPs [21,22]. The protein-protein interaction (PPI) network was constructed using STRING v.11.5 (https://string-db.org) and GeneMANIA (http://genemania.org/).

### 2.7 Animals and in vivo study design

A total of 48 female Wistar rats (8 weeks old; 200-220 g; Janvier Labs) were randomly (random number table) assigned to four groups: sham group (group 1; n = 12), SCI/vehicle group (group 2; n = 12), inducer group (group 3; n = 12) and Mi group (group 4; n = 12) (Fig. 1). Individual animals were identified by tail marking using a non-toxic permanent marker, a commonly used method for rodent identification [23]. During the study, three animals were housed in one cage with a 12:12 h light/dark cycle at 26 °C and with food and water ad libitum. All surgeries and outcome assessments were blinded, all animal experiments were conducted in accordance with the ARRIVE guidelines and the Guide for the Care and Use of Laboratory Animals, and all experimental protocols were approved by the Animal Care Committee of the federal government of Baden-Württemberg, Germany (ethical approval code G-285/19).

**Fig. 1:**
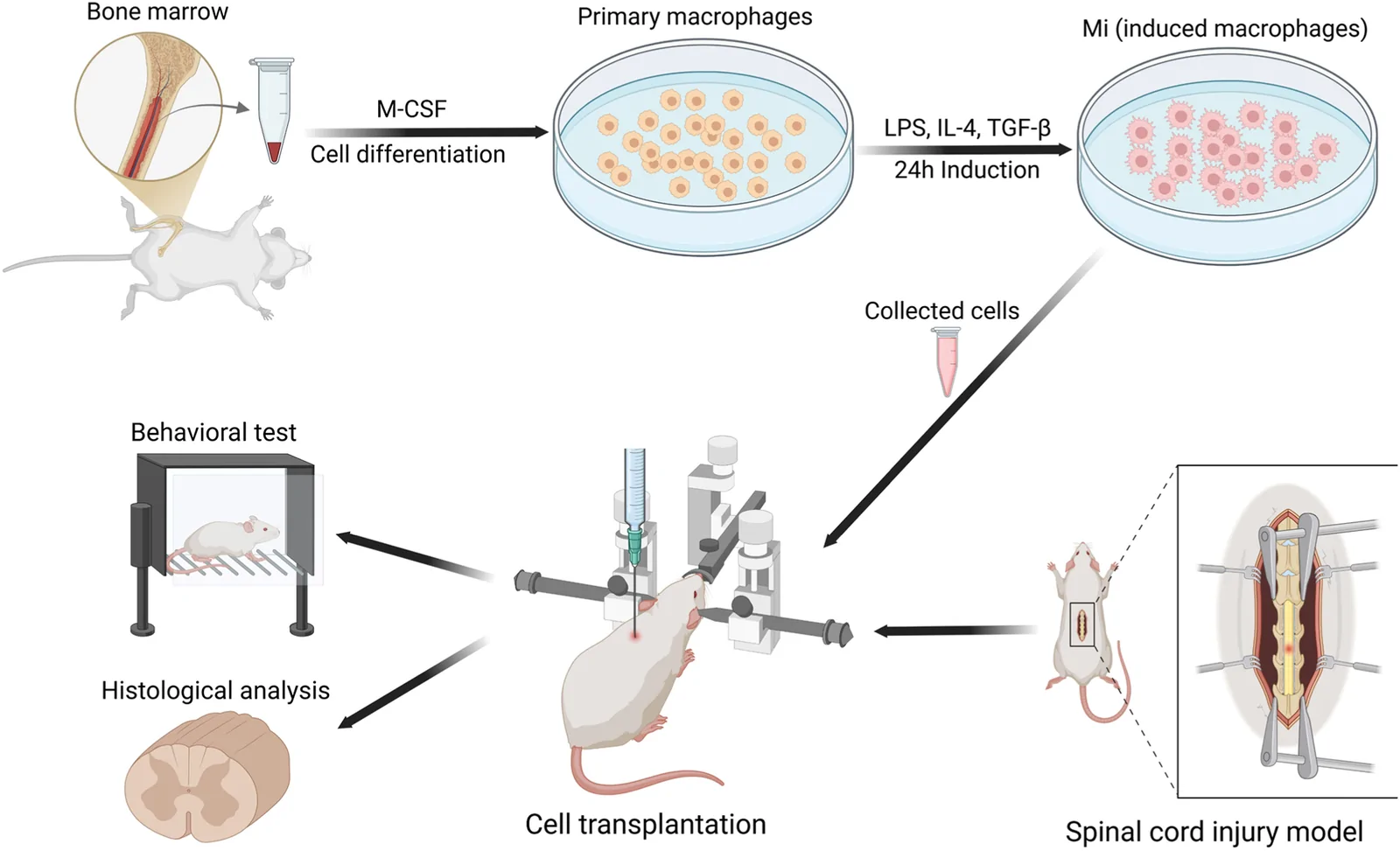
Overview of the experimental workflow from the generation of primary macrophages and their induction in vitro to the transplantation of induced macrophages (Mi) into the lesioned spinal cord after thoracic spinal cord injury (SCI) in vivo (BioRender). Behavioural assessments were performed at baseline and at 3, 7, and 14 days after SCI; tissue collection was performed at 3 or 14 days after SCI depending on the analysis.

### 2.8 Spinal cord injury model and stereotactic transplantation

Animals in groups 2-4 underwent a thoracic contusion–compression SCI using a 28-g modified aneurysm clip (Fehlings’ Laboratory, Toronto, Canada) at the T9 level, following a protocol similar to a previously described SCI protocol [24,25]. Briefly, a laminectomy was performed at T9, and the clip was placed around the exposed spinal cord, snap shut, and maintained in position for 1 min. Animals in the sham group (group 1) received only a T9 laminectomy without clip application. Immediately following injury, stereotactic transplantation of cells (Mi), inducers (LPS, IL-4, and TGF-β), or vehicle (PBS) was performed using a 10 μL Hamilton syringe (Model 701 RN SYR, Hamilton Company, Bonaduz, Switzerland) fitted with a 35g beveled NanoFil microneedle (NF35BV-2, World Precision Instruments, Berlin, Germany). Thereby, group 4 (Mi group) received 10 μL of PBS containing 1 × 10^6^ Mi, injected into the spinal cord at the level of the lesion epicenter at two lateral sites - 1 mm from the midline on each side - with 5 μL delivered per site. The microneedle was inserted to a depth of 1.5 mm from the dorsal surface, and each injection was administered at a rate of 0.5 μL/min. In the same manner, the vehicle group (group 2) received 10 μL of PBS alone. The inducer group (group 3) received 10 μL of PBS containing 16.65 μg LPS, 133.2 ng IL-4, and 66.6 ng TGF-β. The inducer group was included to control for the local effects of LPS, IL-4, and TGF-β in the absence of cell transplantation, allowing discrimination between cytokine- and Mi cell–mediated effects; The doses of LPS, IL-4, and TGF-β used in the inducer group were empirically selected based primarily on reported local cytokine/LPS administration ranges in previous rodent CNS disease models, together with the relative concentrations of the inducing factors used in vitro Mi induction protocol and preliminary dose-selection considerations [11,26,27]. Postoperative care included administration of analgesics - Meloxicam (2 mg/kg, Boehringer Ingelheim, Germany) and Buprenorphine (0.05 mg/kg, Bayer, Leverkusen, Germany) - twice daily for seven days. For the first week post-surgery, prophylactic antibiotics (2.5% Enrofloxacin, Bayer AG, Leverkusen, Germany) were added to the drinking water, which was refreshed every two days. The bladders were manually expressed twice daily until spontaneous voiding resumed. Only animals that showed typical signs of SCI, including complete hindlimb paralysis or dragging after recovery from anaesthesia and visible spinal cord compression after clip removal, were included in the study. Exclusion criteria were predefined before data analysis and consistently applied. Animals were excluded under the following conditions: (1) technical failure during surgery; (2) failure to develop complete hindlimb paralysis after recovery from anaesthesia; (3) rapid or severe deterioration in health status based on daily scoring; (4) major post-operative complications, such as wound dehiscence or infection despite appropriate intervention; and (5) mortality directly related to the surgical or trauma procedure before data collection. Animals reaching predefined humane endpoints were euthanized under veterinary supervision and excluded from the final analysis. During the study, early deaths attributed to thoracic SCI occurred in groups 2, 3, and 4 (n = 1, 2, and 2, respectively). These animals were replaced before data collection to preserve the predetermined final sample size. All replacement animals underwent the same randomisation, surgical procedure, postoperative care, blinded assessment, and predefined exclusion criteria as the initially assigned animals. The final analyses shown in the figures were based on n = 6 independent biological replicates per group for the corresponding histological and behavioural assessments.

### 2.9 Western blot

Western blot analysis was performed using standard procedures. Equal amounts of protein were separated by SDS-PAGE and transferred onto polyvinylidene fluoride membranes (Millipore, Billerica, USA). Membranes were blocked with 5% non-fat milk in TBST for 1 h at room temperature and incubated overnight at 4 °C with the following primary antibodies: anti-HK1 (1:8000, 19662-1-AP, Proteintech), anti-OPA1 (1:8000, 27733-1-AP, Proteintech), anti-AIF (1:4000, 17984-1-AP, Proteintech), and anti-GAPDH (1:2000, 60004-1-Ig, Proteintech). Membranes were cut according to the expected molecular weights before antibody incubation. After washing, membranes were incubated with HRP-conjugated secondary antibodies (1:20000, RGAR001, Proteintech) for 1 h at room temperature. Protein bands were visualised using enhanced chemiluminescence (ECL) and quantified by densitometry using ImageJ software.

### 2.10 Immunohistochemistry staining

Fourteen days after SCI or sham surgery, animals were euthanized with 5% isoflurane and transcardially perfused with PBS followed by 4% PFA. Spinal cords were post-fixed, cryoprotected in 30% sucrose, and 10-mm segments centred on the lesion were embedded in Tissue-Tek O.C.T. Compound (Tissue-Tek O.C.T. Compound, Sakura Finetek Europa B.V., Alphen aan den Rijn, Netherlands). Transverse sections (30 μm) were cut using a Leica CM3050S cryostat (Leica Biosystems, Nussloch, Germany). Nine serial cross-sections were collected symmetrically at defined distances from the lesion epicenter (0, ±480, ±960, ±1440, and ±1920 μm), covering a 4-mm segment. The −480 to +480 μm region was defined as the epicenter zone. Sections were air-dried and stored at −80 °C until immunohistochemistry (IHC) staining.

To this end, a previously described standard protocol was used [28]. In short, cross-sections were incubated in a blocking solution (0.3% Triton X-100 and 2% donkey and/or goat serum in 1×PBS) for 1 h and then incubated with the following primary antibodies overnight: anti-CD206 (1:1000, HS-488003, Synaptic Systems), anti-GFAP (1:500, 173004, Synaptic Systems), anti-IBA1(1:250, NB100-1028, Novus Biologicals), anti-CSPG (1:500, 453004, Synaptic Systems), anti-Olig2 (1:200, AF2418-SP, R&D system), anti-MBP (1:200, 78896, Cell signaling), anti-NeuN (1:800, 266004, Synaptic Systems), anti-Cleaved Caspase3(1:200, 9664, Cell signaling), anti-CD86 (1:300, BS-1035R, Bioss). The cross-sections were then incubated with secondary antibodies (1:500; A-31572, Invitrogen; 706-605-148, Jackson ImmunoResearch; A32814, Invitrogen; 711-605-152, Jackson ImmunoResearch; A-11073, Invitrogen) and DAPI (1:1000; #6335.1, Carl Roth, Germany) for 1 h and sealed with mounting medium before image taking and analysis.

### 2.11 Microscopy and image analysis

ICC fluorescence images were acquired using a confocal laser scanning microscope (LSM 700; Carl Zeiss) with ZEN software (Carl Zeiss Microscopy GmbH, Jena, Germany) in 8-bit format. Immunohistochemically stained spinal cord sections were scanned using an automated slide scanner (Axio Scan.Z1, Carl Zeiss Microscopy GmbH, Germany) at 20× magnification for quantitative analysis. Image analysis for the ICC and IHC staining was performed using ImageJ (National Institute of Health) and the ZEISS microscope software (ZEN 3.9, Carl Zeiss Microscopy GmbH, Germany). Astrogliosis, tissue scarring, and myelination were quantified by measuring the integrated fluorescence intensity (relative fluorescence units, RFU) of glial fibrillary acidic protein (GFAP), chondroitin sulfate proteoglycans (CSPG), and myelin basic protein (MBP) on nine spinal cord cross-sections per staining group. Cyst area was determined on nine GFAP-stained sections. Values were averaged per animal and compared between groups.

Cell quantification in vitro and in vivo was performed using a previously described semi-automatic algorithm [24]. In ICC experiments, M1 macrophages were defined as iNOS+/DAPI+, M2 macrophages as Arg1+/DAPI+, and neurons as MAP2+/DAPI+. In IHC analyses, IBA1^+^/CD86^+^ and IBA1^+^/CD206^+^ lesion-associated phagocytes were quantified as pro-inflammatory-associated and anti-inflammatory-associated phagocyte populations, respectively, apoptotic cells as Cleaved Caspase3+/DAPI+, apoptotic neurons as NeuN+/Cleaved Caspase3+, and oligodendrocytes as Olig2+/DAPI+. Positive cells were normalized to the region of interest (ROI) area and expressed as cell density (cells/mm^2^), averaged per well or animal, and compared between groups.

### 2.12 Behavioral analysis

Hindlimb locomotor function was evaluated using the Basso–Beattie–Bresnahan (BBB) open-field score (0–21). Animals were video-recorded for 4 min at each time point, and scores were independently assigned by two blinded observers; the mean value was used for analysis [29].

Fine sensorimotor coordination was assessed using the Gridwalk test on a 1-m irregular grid walkway. Hindlimb slips were counted as errors across four trials per animal by two blinded observers, and mean error numbers were used for analysis [30]. Gait analysis was performed using the CatWalk XT system (version 10.5; Noldus Information Technology, Netherlands). Animals traversed an illuminated glass walkway, and at least three compliant runs per time point were recorded and analysed. Automated classifications were verified by a blinded observer [31]. The BBB score was assessed at baseline and on days 3, 7, and 14 after injury. The Gridwalk and CatWalk XT tests were conducted at baseline and at 7 and 14 days after injury.

### 2.13 Model development and correlation analysis

Spinal cord injury specimens were subjected to quantitative histological and functional assessment (see above). For each animal, continuous functional recovery was measured using the BBB locomotor score at day 14, and a binary recovery outcome was defined as BBB≥8 versus < 8 for logistic regression analyses. All data preprocessing and statistical analyses were performed in R 4.4.2 (2024-10-31 ucrt) on a Windows 11 x64 platform using the tidyverse suite for data wrangling, plotROC and patchwork for receiver operating characteristic (ROC) visualization, and pROCroc with DeLong's test for area under the curve (AUC) comparisons. Univariate linear regression models were fit to each biomarker against the continuous BBB score, while univariate logistic models assessed each marker's predictive performance for the dichotomized outcome. Model performance was evaluated by adjusted R^2^ for linear models and AUC for logistic models. Comparison of information criteria (AIC/BIC) was then used to identify top-performing predictors, with final results visualised in facetted ROC plots and summarized in tables. Correlation analysis was performed using ggpairs to visualise variable distributions and pairwise Pearson correlations across groups.

### 2.14 Statistical analysis

Unless otherwise specified, all data are presented as the mean ± standard deviation (SD). Normality was tested by the Shapiro–Wilk test. For two-group comparisons, an unpaired t-test was used. For the comparison of multiple groups after ICC, qPCR, phagocytosis assays, and IHC, repeated measures of one-way analyses of variance (ANOVAs) followed by Bonferroni post hoc tests were applied. For the comparison of multiple groups and timepoints, repeated measures of two-way ANOVAs followed by Bonferroni post hoc tests were used. For proteomic analysis, a one-way ANOVA was performed with Benjamini-Hochberg correction to control for false discovery rates. This was followed by pairwise comparisons using Tukey's HSD test. Proteins were considered significant if their relative intensity showed a fold-change of ± 2, and the corresponding *P*-values from Tukey's HSD test and the BH-corrected ANOVA were both ≤ 0.05. For all other analyses, statistical significance was defined as *P* < 0.05. Statistical analyses were performed using GraphPad Prism (version 9.2; GraphPad Software, California, USA) and R 4.4.2.

## 3 Results

### 3.1 LPS, IL-4, and TGF-β treatment increases anti-inflammatory marker expression in Mi

To determine the effects of LPS, IL-4, and TGF-β treatment on the anti-inflammatory phenotype of Mi, we analysed the expression of key markers (Fig. 2A). After 24 h of incubation, quantitative PCR revealed that the expression of anti-inflammatory markers Arginase 1 (Arg1) and IL-10 was significantly upregulated in Mi compared to resting BMDMs (*P* = 0.0002 and *P* = 0.008, respectively) (Fig. 2B and C). In contrast, the mRNA expression levels of pro-inflammatory markers iNOS, IL-1β, TNF-α, and IL-12 were markedly lower in Mi than in M1-polarised BMDMs (*P* < 0.0001, *P* < 0.0001, *P* = 0.006, *P* < 0.0001, respectively) (Fig. 2D–G). Immunofluorescence (IF) analysis further confirmed that the iNOS protein expression was minimal in Mi and comparable to that in resting BMDMs (*P* > 0.99). In contrast, the expression level of Arg1 protein in Mi was higher than that in M2-polarised macrophages (*P* < 0.001), consistent with the qPCR results (Fig. 2H–K).

**Fig. 2:**
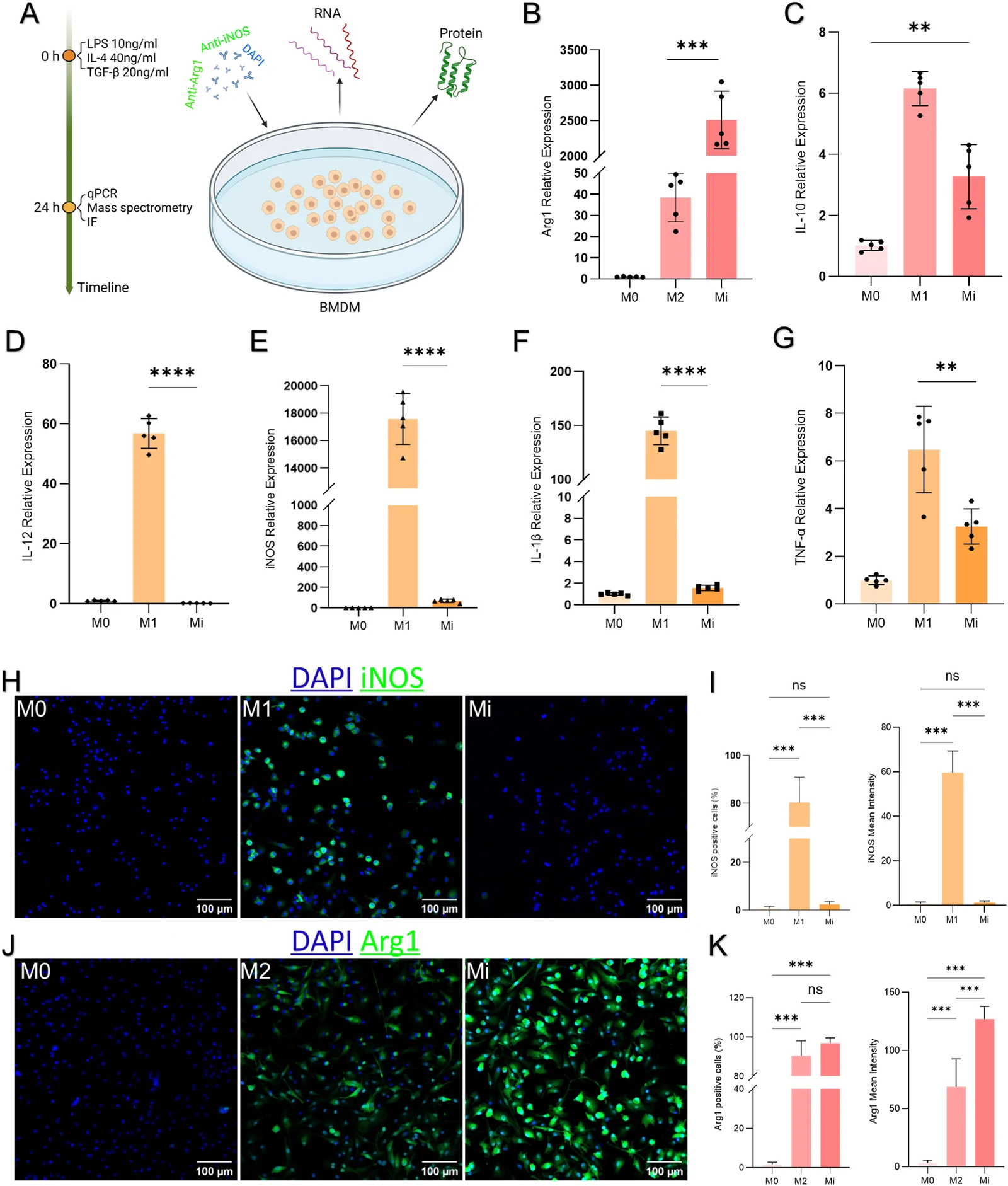
Molecular signatures of induced macrophages (Mi). (**A**) Schematic illustration of the experimental workflow for Mi induction and phenotypic analysis. Bone marrow-derived macrophages (BMDMs) were left untreated as M0 macrophages or stimulated with defined cytokine combinations to generate M1, M2, or Mi macrophages. Mi macrophages were induced by combined stimulation with LPS, IL-4, and TGF-β for 24 h, followed by qPCR and immunocytochemical analyses. Created with BioRender. (**B-G**) Quantitative PCR (qPCR) analysis of macrophage phenotypes (M0, M1, M2, and Mi) showing significantly elevated expression levels of anti-inflammatory markers (Arg1, IL-10) and reduced levels of pro-inflammatory markers (IL-1β, IL-12, TNF-α, and iNOS) in Mi macrophages. (**H, I**) Representative confocal images and quantification of iNOS immunofluorescence in M0, M1, and Mi macrophages. iNOS is shown in green, and nuclei are counterstained with DAPI in blue. Scale bar = 100 μm. (**J, K**) Representative confocal images and quantification of Arg1 immunofluorescence in M0, M2, and Mi macrophages. Arg1 is shown in green, and nuclei are counterstained with DAPI in blue. Scale bar = 100 μm. Data are presented as mean ± SD; n = 5 independent experiments/group. For qPCR analyses, unpaired Student's t-test was used for two-group comparisons. For immunofluorescence quantification, one-way ANOVA followed by Bonferroni post hoc test was used. ns: not significant, *P < 0.05, **P < 0.01, ***P < 0.001, ****P < 0.0001).

### 3.2 Proteomic analysis suggests a repair-associated profile in Mi macrophages

To further explore the molecular phenotype of Mi, we conducted a proteomic analysis comparing Mi to resting BMDMs. Proteomic analysis identified 122 proteins downregulated and 134 proteins upregulated in the Mi macrophage line compared to resting BMDMs. The top 25 differentially expressed proteins (DEPs) are shown in the heatmap, based on the magnitude of their expression changes, and include Ripk2, Nolc1, and Wdfy3 (Fig. 3A and B). GO and KEGG analyses indicated that DEPs in Mi macrophages were mainly associated with lysosomal activity, metabolism, vesicular processes, and immune responses, suggesting molecular features distinct from resting macrophages (Fig. 3C and D). Specifically, the DEPs were also markedly enriched in biological processes linked to IL-10 production, phagocytosis, tissue remodelling, oligodendrocyte development, axonal ensheathment, and myelin formation (Fig. 3E). These enrichment patterns suggest that Mi macrophages may acquire a repair-associated molecular profile.

**Fig. 3:**
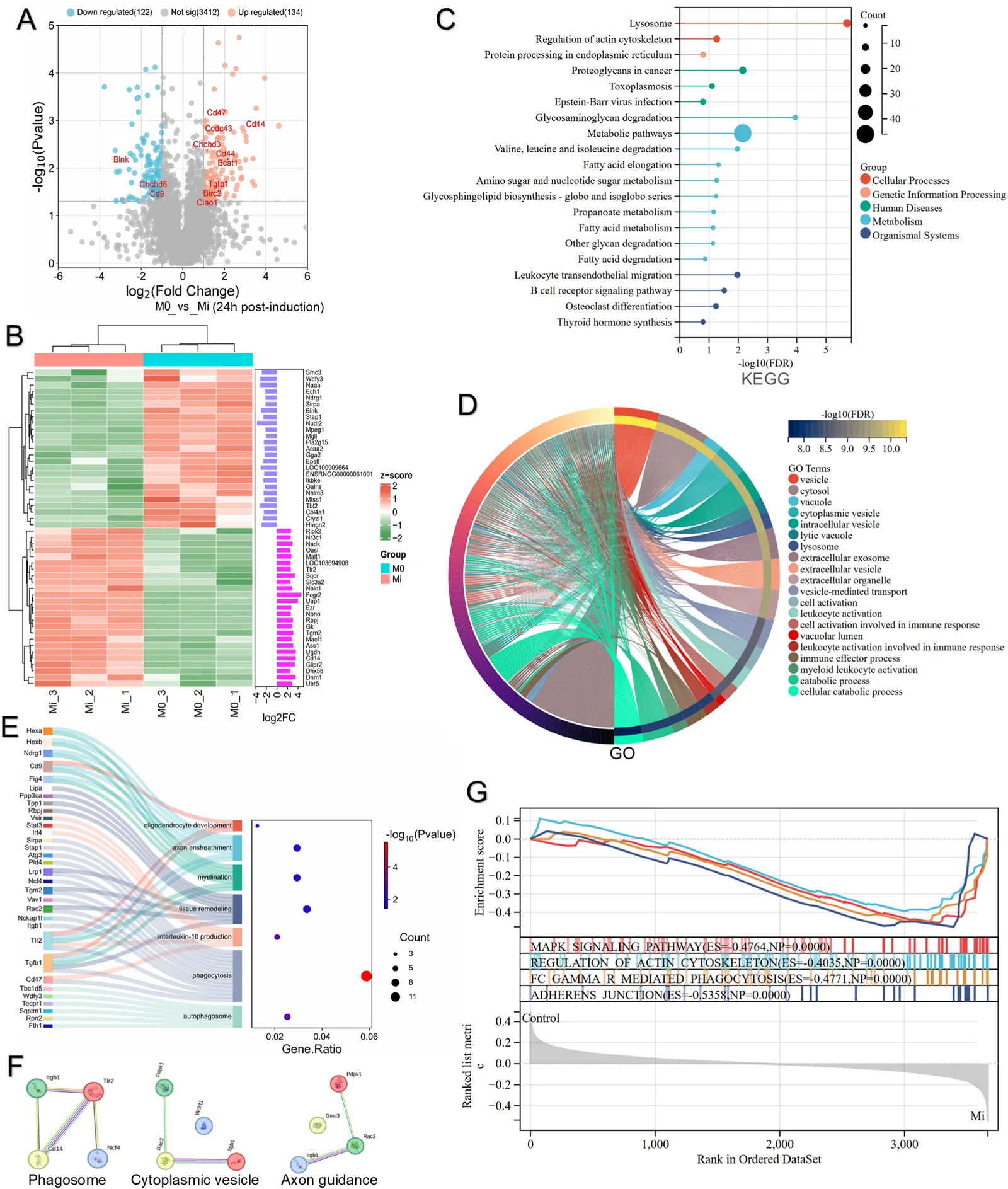
Proteomic profiling and pathway enrichment of Mi macrophages. (**A**) Volcano plot showing the differentially expressed proteins (DEPs) between Mi and M0 macrophages (n = 3/group, *P* < 0.05, |Log2(FC)| > 1). A total of 122 proteins were downregulated and 134 proteins were upregulated in Mi macrophages compared with M0 macrophages. (**B**) Heatmap showing the top 25 DEPs ranked by expression change between Mi and M0 macrophages. (**C**) KEGG pathway enrichment analysis of DEPs showing the top 20 enriched pathways ranked by false discovery rate (FDR). (**D**) Gene Ontology (GO) enrichment analysis of DEPs showing the top 20 enriched terms across biological process, cellular component, and molecular function categories ranked by FDR. (**E**) Enrichment analysis of DEPs revealed significant enrichment in biological processes related to IL-10 production, phagocytosis, tissue remodelling, oligodendrocyte development, axon ensheathment, and myelination, suggesting a potential role in tissue repair and regeneration. (**F**) Protein–protein interaction (PPI) network analysis performed using STRING, showing enriched clusters of upregulated proteins associated with axon guidance, cytoplasmic vesicles, and phagosome-related pathways. Three representative functional subsets of the PPI network are shown. (**G**) Gene set enrichment analysis (GSEA) comparing Mi and M0 macrophages, showing enriched pathways related to MAPK signalling, Fc gamma receptor-mediated phagocytosis, adherens junction, and regulation of the actin cytoskeleton.

To investigate functional modules within these 256 DEPs, a protein–protein interaction (PPI) network was constructed (Fig. S1C). This analysis showed that Mi upregulated pathways related to axon guidance, cytoplasmic vesicles, phagosome activity, and thereby enhanced neural repair and phagocytic capacity (Fig. 3F). Further pathway analysis revealed upregulation of proteins involved in protein processing, mitochondrial (chondriosome) functions, and key signalling pathways such as the cAMP (Atp1b3, Gnai3, Rac2, Acox1, Vav1) and Rap1 signalling pathways (Fig. S1D). These pathways are known to regulate macrophage polarisation, phagocytosis, inflammatory responses, angiogenesis, and tissue repair and regeneration. GSEA revealed that four pathways - MAPK signalling, Fc gamma receptor–mediated phagocytosis, adherens junction, and regulation of the actin cytoskeleton - were significantly enriched in the induced macrophages (Mi) (Fig. 3G). Together, these pathways suggest that Mi may support inflammatory modulation, phagocytic clearance, cytoskeletal organisation, and tissue remodelling. In summary, Mi macrophages display a repair-associated proteomic profile consistent with an immunomodulatory and pro-reparative bias.

### 3.3 Mi exhibit robust phagocytotic capacity without inducing neuronal cytotoxicity

**Fig. 4:**
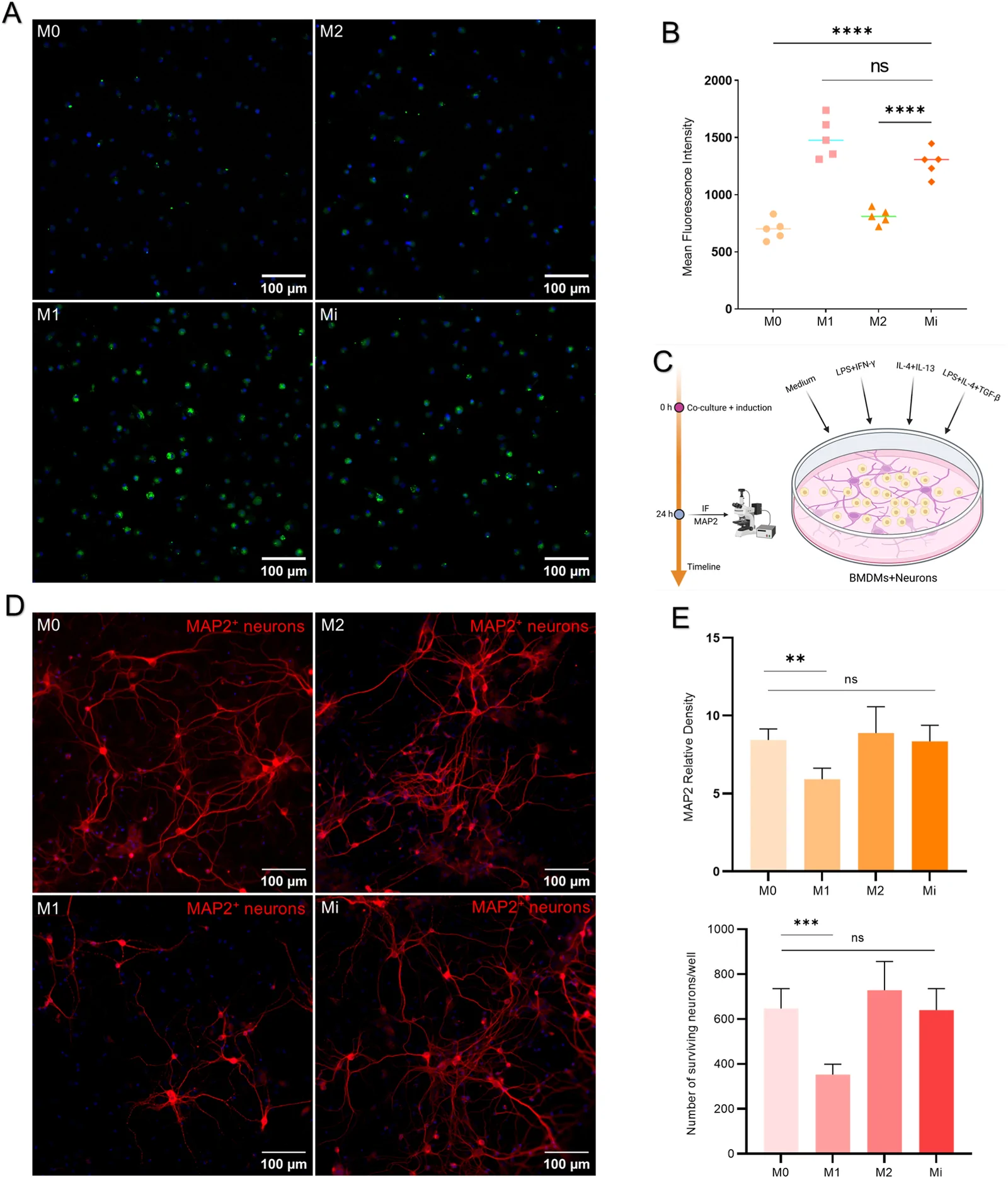
Phagocytosis assay of macrophages with different phenotypes and their co-culture with neurons. (**A**) Representative fluorescence images showing microsphere uptake by bone marrow-derived macrophages (BMDMs) with different phenotypes (M0, M1, M2, and Mi). Scale bar = 100 μm. (**B**) Quantification of phagocytic activity based on mean fluorescence intensity of engulfed microspheres. (**C**) Schematic illustration of the macrophage–neuron co-culture assay. Differently induced BMDMs were co-cultured with primary neurons for 24 h. Created with BioRender. (**D**) Representative confocal images of primary neurons after co-culture with macrophages under the indicated conditions. Neurons were immunostained for MAP2 (red). Scale bar = 100 μm. (**E**) Quantitative analysis of relative MAP2 immunofluorescence intensity and number of neurons demonstrated significantly reduced levels in the M1 macrophage group compared to the other groups. In contrast, neurons co-cultured with Mi (LPS/IL-4/TGF-β-stimulated) macrophages exhibited MAP2 immunointensity and number of neurons comparable to the M0 and M2 groups, with no significant decrease in neuronal preservation observed in the Mi group. Data are expressed as mean ± SD (n = 5/group; one-way ANOVA followed by Bonferroni post hoc test. ns: not significant, *P < 0.05, **P < 0.01, ***P < 0.001, ****P < 0.0001).

To assess the phagocytic activity of Mi, we performed fluorescent microsphere uptake assays. As shown in Fig. 4A and B, treatment with LPS, IL-4, and TGF-β significantly enhanced the uptake of microspheres by BMDMs. Mi demonstrated a significantly greater phagocytic capacity than both resting (M0) and M2 macrophages (*P* < 0.0001 and *P* < 0.0001, respectively). Additionally, the phagocytic capacity of Mi was not significantly lower than that of M1 (*P* = 0.054).

This enhanced phagocytic capacity may support debris clearance during the early phase of injury.

Additionally, we investigated whether the inclusion of LPS in the Mi induction protocol (LPS/IL-4/TGF-β) would result in neuronal death when co-cultured with macrophages (Fig. 4C). Compared with the M0 group, co-culture with M1 macrophages significantly reduced the number of MAP2^+^ neurons and neurite density (*P* = 0.0002 and *P* = 0.0086, respectively), indicating neuronal loss under this condition. In contrast, co-culture with Mi did not result in neuronal death or reduction in neurite density (*P* > 0.99 and *P* = 0.9987, respectively) (Fig. 4D and E) [32].

These findings suggest that Mi macrophages retain strong phagocytic activity without inducing detectable neuronal toxicity under these co-culture conditions.

### 3.4 Mi transplantation induces an anti-inflammatory shift in lesion-associated phagocytes

**Fig. 5:**
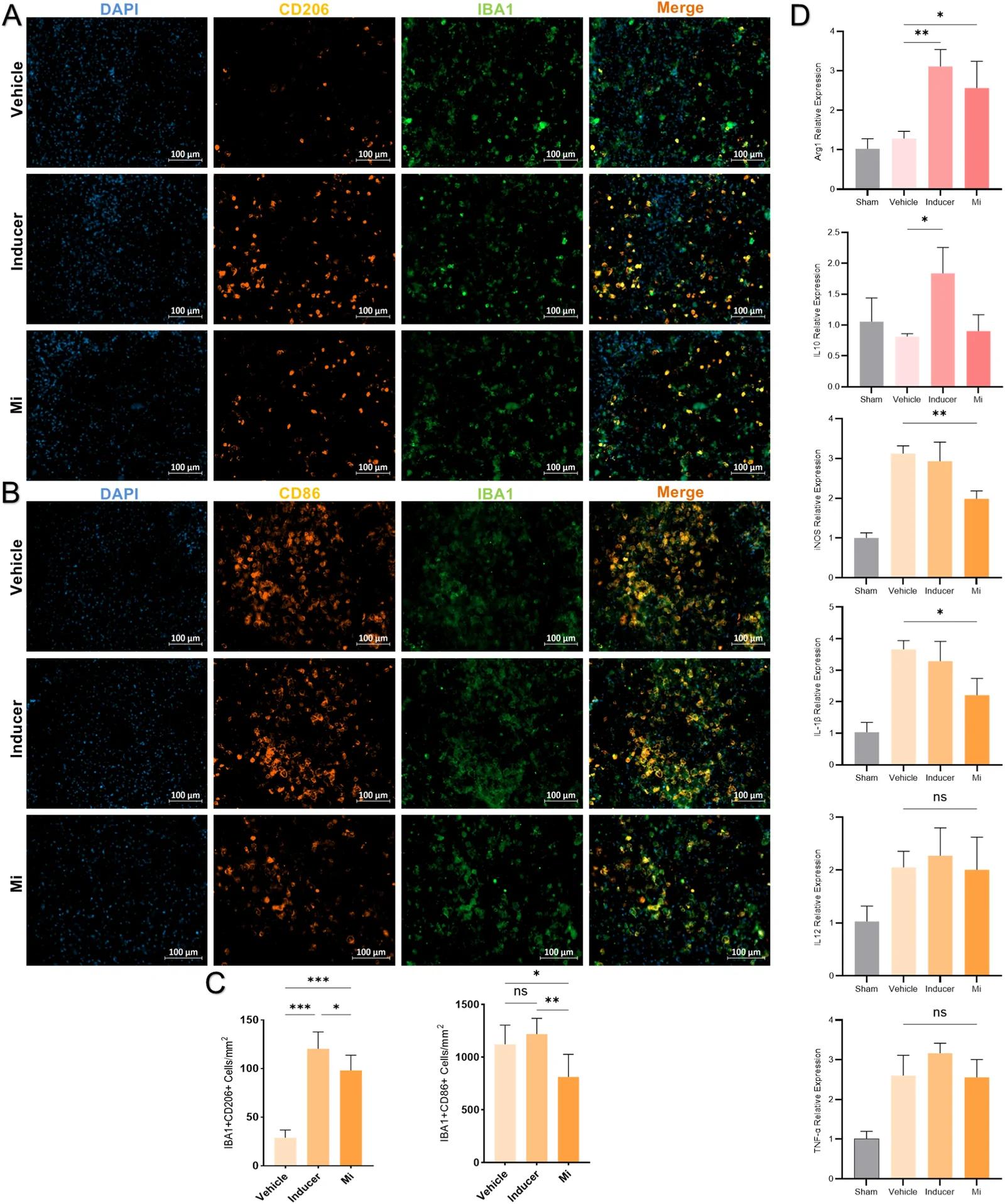
Mi transplantation modulates lesion-associated inflammatory phagocytes and inflammatory gene expression after SCI. (**A**) Representative immunofluorescence images showing IBA1^+^/CD206^+^ cells in the injured spinal cord 14 days after SCI. Scale bar = 100 μm. (**B**) Representative immunofluorescence images showing IBA1^+^/CD86^+^ cells in the injured spinal cord 14 days after SCI. Scale bar = 100 μm. (**C**) Quantification of IBA1^+^/CD206^+^ and IBA1^+^/CD86^+^ cell densities in the lesion area. (**D**) Relative mRNA expression levels of Arg1, IL-10, iNOS, IL-1β, IL-12, and TNF-α in spinal cord tissues collected three days after SCI. Relative mRNA expression was normalized to Actb. Data are expressed as mean ± SD (n = 6/group; one-way ANOVA followed by Bonferroni post hoc test. ns: not significant, *P < 0.05, **P < 0.01, ***P < 0.001).

To assess whether Mi transplantation modulates the inflammatory environment after SCI, we analysed lesion-associated IBA1^+^ phagocytes 14 days after injury. Notably, both the inducer group and the Mi group exhibited a significantly higher density of anti-inflammatory-associated IBA1^+^/CD206^+^ cells compared to the vehicle group (*P* < 0.001 for both comparisons) (Fig. 5A and C). The Mi group also displayed a significantly lower density of pro-inflammatory-associated IBA1^+^/CD86^+^ cells compared to the vehicle group (*P* = 0.03) and the inducer group (*P* = 0.005) (Fig. 5B and C). In contrast, there was no significant difference in the density of IBA1^+^/CD86^+^ cells between the inducer and vehicle groups (*P* > 0.99).

We next examined inflammatory gene expression in spinal cord tissues collected three days after SCI. Compared to the vehicle group, both the inducer and Mi groups showed significantly elevated expression of the anti-inflammatory marker Arg1 (*P* = 0.005, *P* = 0.04, respectively) (Fig. 5D). The inducer group also exhibited increased expression of IL-10 (*P = 0.02*). In contrast, only the Mi group showed a significant decrease in mRNA levels of the pro-inflammatory markers iNOS and IL-1β (*P* = 0.007, *P* = 0.03, respectively) (Fig. 5D).

Together, these findings suggest that Mi transplantation promotes an anti-inflammatory shift in lesion-associated phagocytes and suppresses selected pro-inflammatory markers during the early post-injury period.

### 3.5 Mi transplantation improves perilesional histopathological outcomes

To assess the impact of Mi transplantation or inducer treatment on the preservation of spinal cord tissue 14 days after SCI in vivo, we quantified the cyst and total spinal cord areas in GFAP-stained cross-sections, extending 1920 μm rostrally and caudally from the lesion epicenter.

**Fig. 6:**
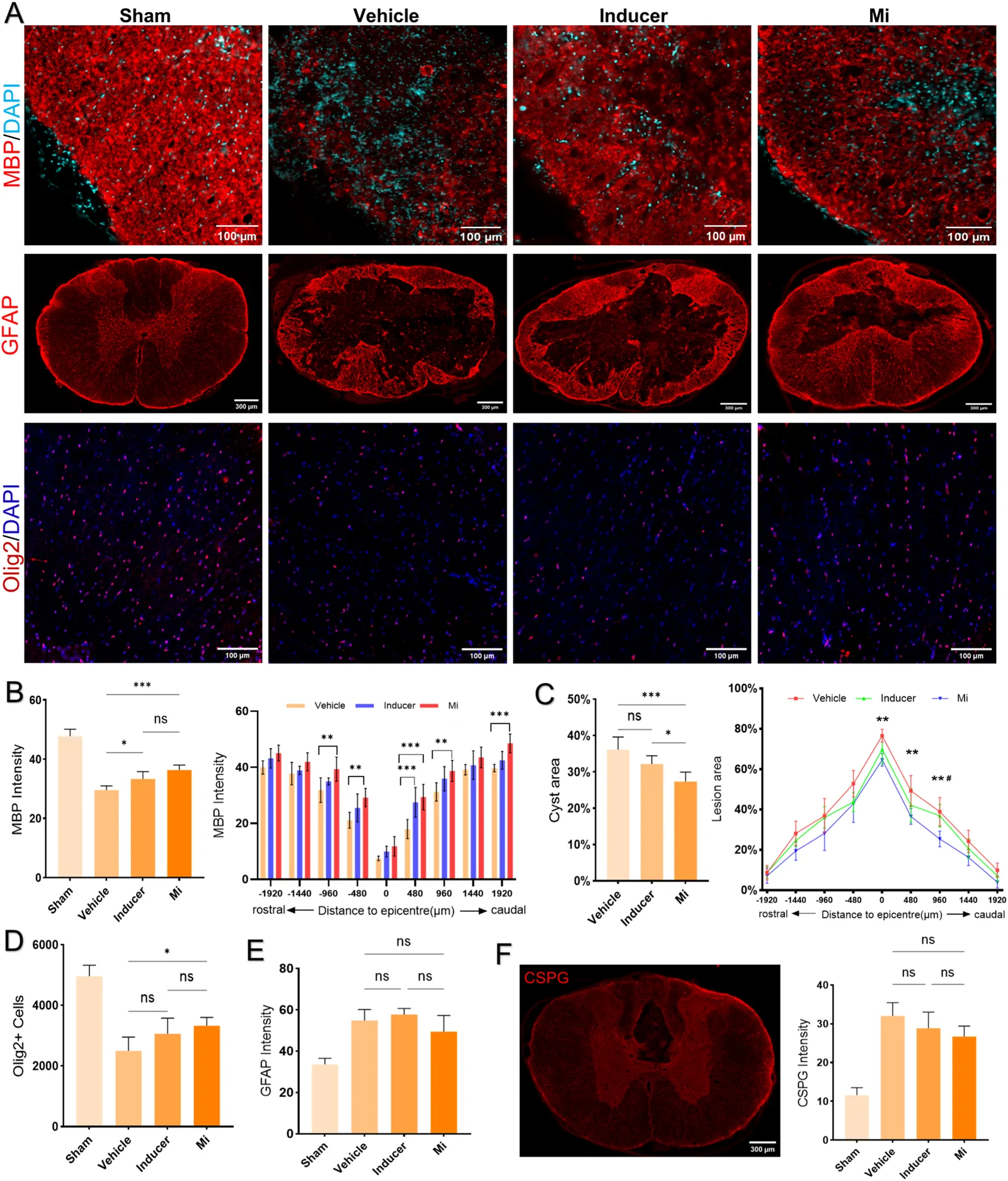
Mi transplantation is associated with improved histopathological outcomes after SCI. (**A, B**) Representative cross-sections of the spinal cord stained for MBP (red) 14 days after SCI. The Mi group exhibited significantly higher MBP immunointensity compared to both the inducer and vehicle groups. The inducer group showed increased MBP immunointensity relative to the vehicle group. Scale bar = 100 μm. (**A, C**) Spinal cord sections stained with GFAP (red) 14 days after SCI. The Mi group exhibited the smallest cyst area compared to all other injured groups. Scale bar = 300 μm. (**A, D**) Representative cross-sectional images of the spinal cord 14 days after SCI, showing Olig2^+^ oligodendrocytes (stained dark red). The Mi group exhibited a significantly greater number of Olig2^+^ oligodendrocytes compared to the vehicle group. Scale bar = 100 μm. (**E, F**) There was no significant difference in CSPG (red) intensity and GFAP^+^ astrocyte immunointensity among the three groups 14 days after SCI. Data are expressed as mean ± SD (n = 6/group; one-way ANOVA followed by Bonferroni post hoc test. ns: not significant, *P < 0.05, **P < 0.01, ***P < 0.001).

The cyst area was significantly smaller in the Mi group compared to both the inducer group (*P* = 0.03) and the vehicle group (*P* < 0.001) (Fig. 6A and C). No significant differences were observed in preserved tissue between the inducer and vehicle groups (*P* = 0.08). Specifically, the Mi group showed smaller cyst areas than the vehicle group at the lesion epicenter and 480 μm and 960 μm caudal to the epicenter (Fig. 6C). These results suggest that Mi transplantation reduces cyst formation and supports early tissue preservation after SCI.

We next assessed myelin-related changes by quantifying MBP immunointensity. MBP immunointensity was significantly higher in both the Mi and inducer groups compared to the vehicle group (*P* < 0.001 and *P* = 0.03, respectively) (Fig. 6A and B, S2B). Increased MBP immunointensity was particularly evident in the Mi group near the lesion epicenter (Fig. 6B). To further explore myelination, we quantified Olig2^+^ oligodendrocytes in each group. Only the Mi group showed a significantly higher number of Olig2^+^ cells compared to the vehicle group (*P* = 0.01) (Fig. 6A and D). These findings suggest that Mi transplantation is associated with improved myelin preservation and increased oligodendrocyte numbers at 14 days after SCI.

We also assessed astrogliosis and tissue scarring by measuring the immunointensity of GFAP and CSPG. No significant differences in astrogliosis were observed among the three injured groups (Fig. 6E). Although the inducer and Mi groups showed a trend toward lower mean CSPG expression compared to the vehicle group, these differences did not reach statistical significance (vehicle vs. inducer, *P* = 0.57; vehicle vs. Mi, *P* = 0.05; inducer vs. Mi, *P* > 0.99) (Fig. 6F). Thus, neither Mi transplantation nor inducer treatment substantially altered astrogliosis or CSPG-associated scarring at this two-week time point.

### 3.6 Mi transplantation is associated with reduced apoptosis and improved motor neuron preservation

**Fig. 7:**
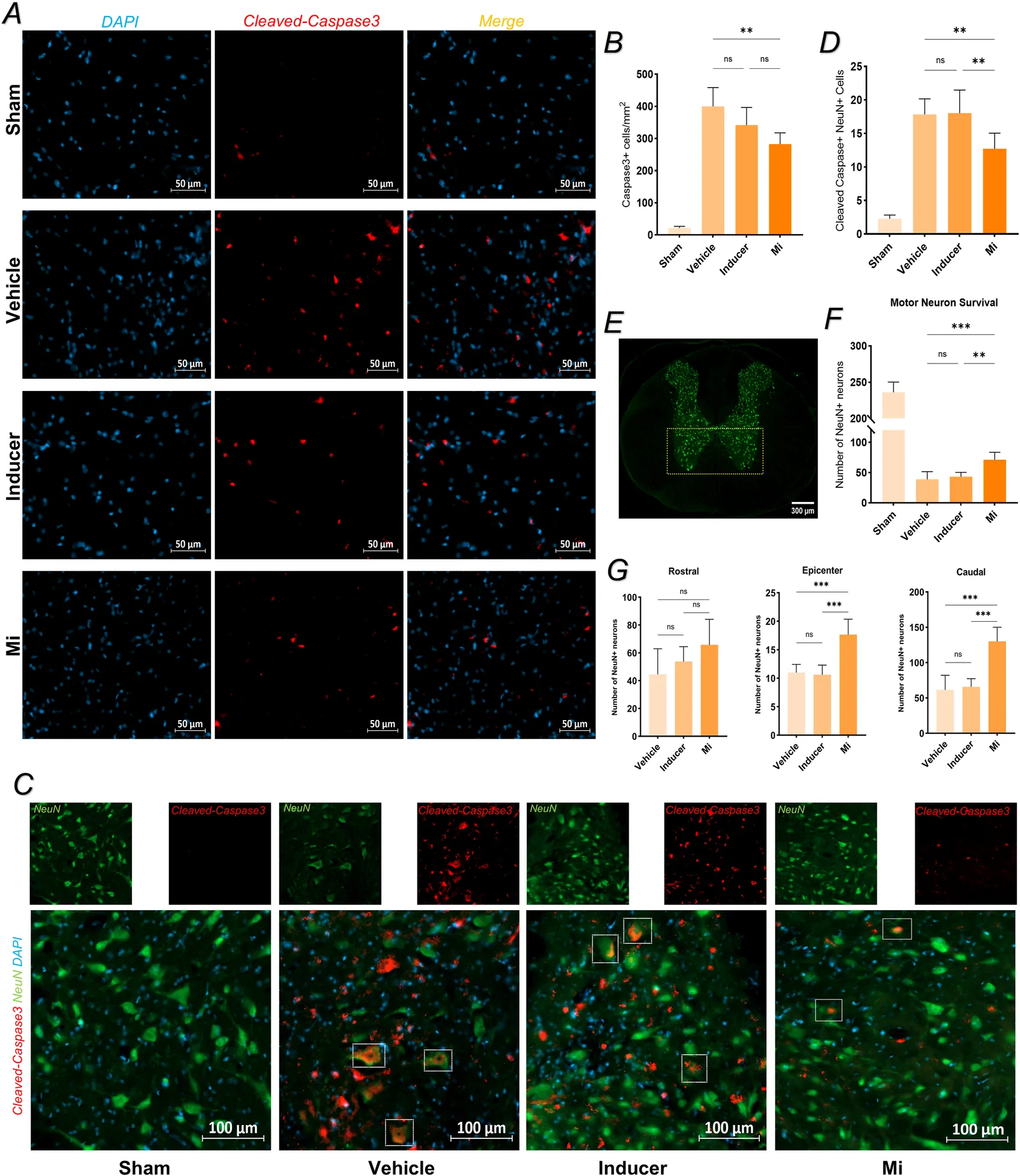
Mi transplantation is associated with reduced apoptosis and improved motor neuron preservation after SCI. (**A, B**) Spinal cord cross-sections stained for Cleaved Caspase-3 (red) to visualise apoptotic cells 14 days after SCI. The density of Cleaved Caspase-3-positive apoptotic cells was significantly reduced in the Mi-treated group compared to the vehicle group. Scale bar = 50 μm. (**C, D**) Spinal cord cross-sections co-stained for Cleaved Caspase-3 (red) and NeuN (green) to identify apoptotic motor neurons. The number of Cleaved Caspase-3^+^/NeuN^+^ cells in the ventral horns was significantly lower in the Mi-treated group compared to the vehicle group 14 days after SCI. Scale bar = 100 μm. (**E, F, G**) NeuN-stained (green) spinal cord cross-sections 14 days after SCI. Quantification of NeuN^+^ cells in the ventral horns showed a significantly higher number of spared motor neurons in the Mi-treated group compared to both the inducer and vehicle groups. Scale bar = 300 μm. Data are expressed as mean ± SD (n = 6/group; one-way ANOVA followed by Bonferroni post hoc test; ns: not significant, *P < 0.05, **P < 0.01, ***P < 0.001).

To evaluate apoptotic cell death after treatment, we quantified Cleaved Caspase-3 expression 14 days after SCI. The density of apoptotic cells was significantly lower in the Mi group compared to the vehicle group (*P* = 0.001). However, no significant differences were observed between the inducer and vehicle groups (*P* = 0.2) or between the inducer and Mi groups (*P* = 0.18) (Fig. 7A and B).

We next assessed motor neuronal apoptosis by double staining for NeuN and Cleaved Caspase-3 in the ventral horns along the rostrocaudal axis. Animals that received Mi transplantation displayed a significant reduction in motor neuronal apoptosis (NeuN^+^/Cleaved Caspase-3^+^ cells) compared to both the vehicle group (*P* < 0.001) and the inducer group (*P* = 0.04) (Fig. 7C and D).

Consistent with the decrease in apoptotic neurons, the overall number of surviving motor neurons (NeuN^+^ cells) in the spinal cords’ ventral horns was significantly higher in the Mi group compared to both the vehicle (*P* < 0.001) and inducer (*P* = 0.004) groups (Fig. 7E and F). Furthermore, the data in Fig. 7G demonstrate that Mi transplantation led to significantly improved motor neuron preservation at the lesion epicenter (*P* < 0.001) and caudal to the epicenter (*P* < 0.001) compared to the vehicle group.

Together, these findings suggest that Mi transplantation is associated with reduced apoptosis and improved motor neuron preservation at the lesion site.

### 3.7 Mi transplantation improves early functional recovery

**Fig. 8:**
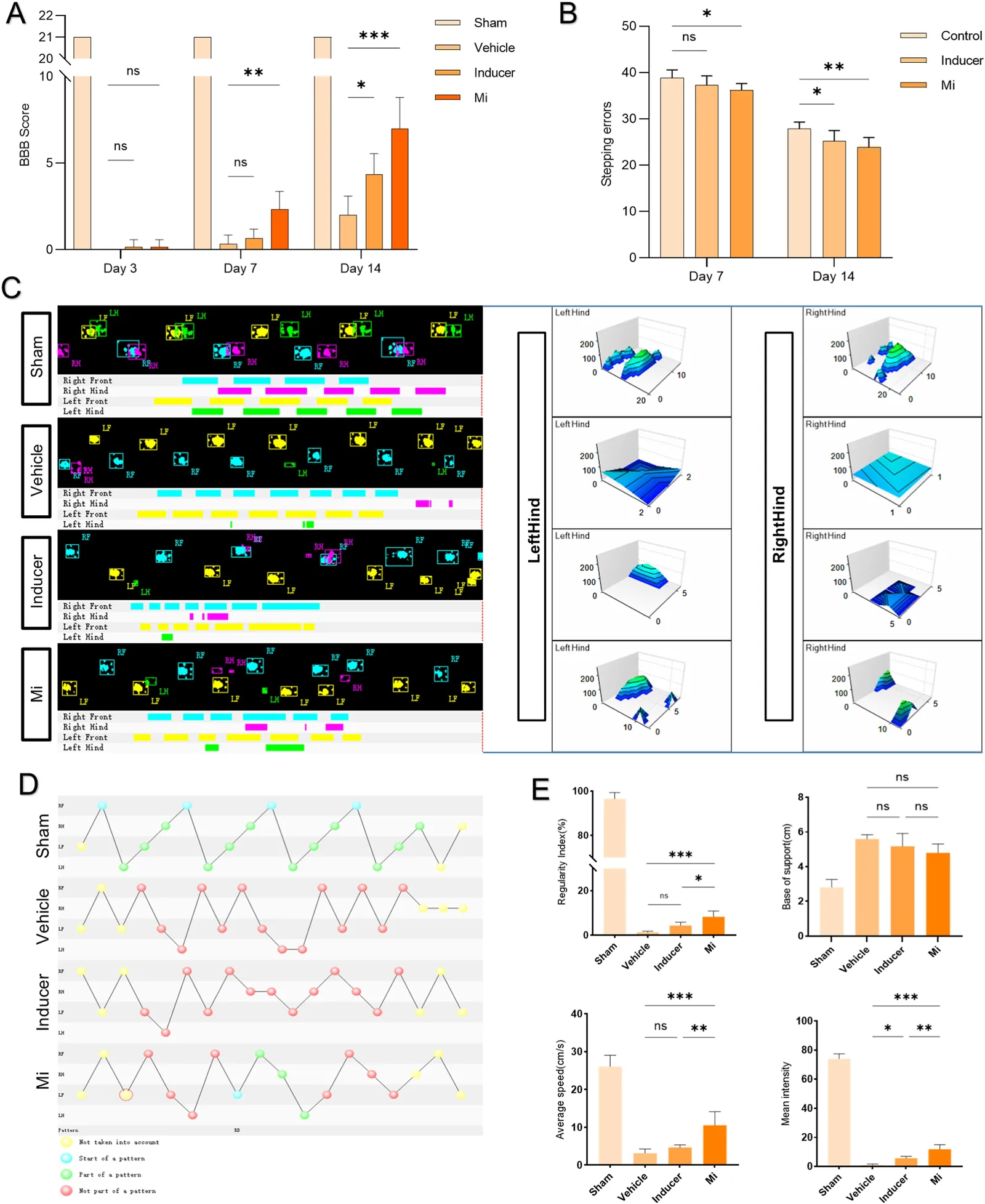
Behavioural assessment of locomotor recovery after SCI. (**A**) Basso–Beattie–Bresnahan (BBB) open-field locomotor scores assessed at baseline and at 3, 7, and 14 days after SCI. The Mi group exhibited the highest BBB scores, indicating the best locomotor recovery, with a significant improvement compared to vehicle-treated animals starting from the 7 days after SCI and continuing through the end of the experiment. At 14 days after SCI, the inducer group also demonstrated significantly better performance than the vehicle group. (**B**) Gridwalk stepping errors assessed at baseline and at 7 and 14 days after SCI. The number of stepping errors in the Gridwalk test was significantly lower in the Mi group compared to the vehicle group at both 7 days and 14 days post-SCI. (**C**) Representative CatWalk paw-print images from each group at 14 days after SCI. (**D**) Representative CatWalk footfall patterns from each group at 14 days after SCI. (**E)** Quantification of CatWalk gait parameters at 14 days after SCI, including regularity index, base of support, walking speed, and mean intensity. Data are expressed as mean ± SD (n = 6/group; BBB scores and Gridwalk errors were analysed using two-way ANOVA followed by Bonferroni post hoc test. CatWalk parameters were analysed using one-way ANOVA followed by Bonferroni post hoc test. ns: not significant, *P < 0.05, **P < 0.01, ***P < 0.001).

Hindlimb locomotor recovery was assessed using the 21-point BBB open-field score. At 7 days after the injury, the Mi group showed a significant improvement compared to the vehicle group (*P* = 0.0062). At the same time, there was no significant difference between the inducer and vehicle groups (*P* = 0.4557) (Fig. 8A). At 14 days after injury, BBB scores were significantly higher in both the inducer group (*P* = 0.0107) and the Mi group (*P* < 0.001) compared to the vehicle group.

The Gridwalk test, which evaluates sensory and motor coordination, showed that stepping errors in the Mi group began to decline as early as 7 days after SCI (*P* = 0.0365). The inducer group did not exhibit significant improvement at this time point (*P* = 0.295) (Fig. 8B). At 14 days after SCI, the Mi group demonstrated the most significant reduction in stepping errors compared to the vehicle group (*P* = 0.0017), followed by the inducer group (*P* = 0.0365). As expected, sham animals exhibited zero or near-zero stepping errors throughout the study period (data not shown).

The CatWalk gait analysis further assessed motor function recovery. No significant difference in the regularity index (RI) was observed between the inducer and vehicle groups during the 14-day observation period after SCI. However, the RI of the Mi group was significantly improved compared to both the vehicle (*P* < 0.001) and inducer groups (*P* = 0.03). The base of support (BOS) did not differ significantly between the injured groups. Animals in the Mi group exhibited faster walking speeds than those in the inducer group (*P* = 0.003) and the vehicle group (*P* < 0.001). At the same time, no significant difference was observed between the vehicle and inducer groups (*P* > 0.99). Moreover, the Mi group showed significantly higher mean intensity (MI) of hindlimb paw placement compared to both the vehicle (*P* < 0.001) and inducer groups (*P* = 0.002), with the inducer group also showing a modest increase in MI compared to the vehicle group (*P* = 0.02) (Fig. 8C, D, and E).

Together, these findings indicate that Mi transplantation improves locomotor and sensorimotor recovery during the early post-injury period.

### 3.8 Correlation and prediction of histological markers for functional recovery

**Fig. 9:**
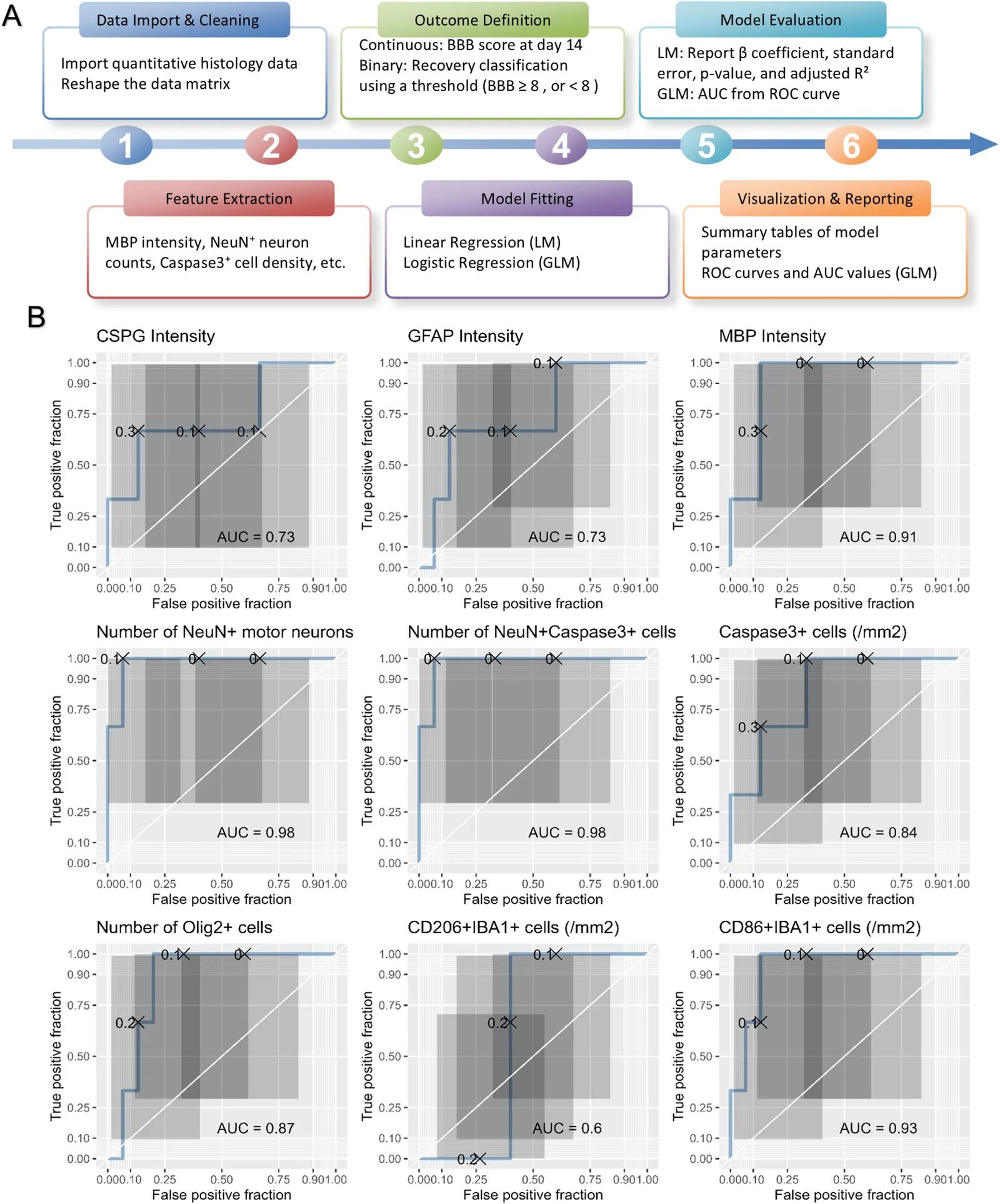
Workflow and ROC curve evaluation of histological markers. (**A**) Overview of the statistical modelling workflow for histological biomarkers. (**B**) Each panel presents the receiver operating characteristic (ROC) curve derived from a univariate logistic regression model, evaluating the ability of a single histological marker to discriminate between animals with good recovery and poor recovery at 14 days after injury. Area under the curve (AUC) values are shown in the lower-right corner of each panel and reflect the discriminative power of each marker. MBP intensity and the number of NeuN^+^ motor neurons showed the highest AUC values, suggesting strong predictive utility. In contrast, markers such as GFAP and CSPG exhibited lower AUCs, indicating limited classification performance.

To examine associations between histological markers and functional recovery after SCI, we performed univariate linear and logistic regression analyses (Fig. 9A, Table S1). The analysed variables included molecular markers (CSPG, GFAP, and MBP intensities), cellular markers (NeuN^+^ motor neurons, NeuN^+^/Cleaved Caspase-3^+^ neurons, Cleaved Caspase-3^+^ cells, Olig2^+^ oligodendrocytes, CD86^+^/IBA1^+^ cells, and CD206^+^/IBA1^+^ cells), and behavioural outcomes (BBB score, Gridwalk errors, and CatWalk parameters) across the sham, vehicle, inducer, and Mi groups. Among the continuous functional outcomes, MBP intensity demonstrated the strongest linear association with 14-day BBB scores (adjusted R^2^ = 0.745, *P* < 0.001), suggesting a close association between MBP immunointensity and locomotor recovery (Table S2). In the logistic regression models predicting binary functional recovery, NeuN^+^Caspase3^+^ cell density yielded the highest discriminative performance (AUC = 0.979), highlighting the prognostic value of neuronal apoptosis (Table S3, Fig. 9B). Other markers such as CD86^+^IBA1^+^ cell density (AUC = 0.931), Olig2^+^ cell count (AUC = 0.871), and Caspase3^+^ cell density (AUC = 0.843) also showed strong predictive value (Fig. 9B). In contrast, CD206^+^IBA1^+^ cells exhibited weak classification performance (AUC = 0.604), suggesting limited relevance in predicting recovery under these experimental conditions.

A pairwise correlation matrix further revealed associations among inflammatory, histological, and behavioural parameters (Fig. S3). MBP intensity and NeuN^+^ motor neuron density were positively correlated with the 14-day BBB score, whereas CD86^+^/IBA1^+^ and Cleaved Caspase-3^+^ cell densities were negatively correlated. These associations were consistent with the best-performing univariate linear and logistic regression models.

Taken together, these findings indicate a multimodal biomarker strategy that integrates myelination (MBP) and neuronal preservation/apoptosis (NeuN^+^ Caspase3^+^) metrics to enhance predictive modelling and translational applicability in Mi-mediated SCI repair. The observed increase in myelination and motor neuron preservation following our treatment provides strong support for the promotion of functional recovery.

### 3.9 Proteomic analysis identifies molecular changes associated with Mi transplantation

**Fig. 10:**
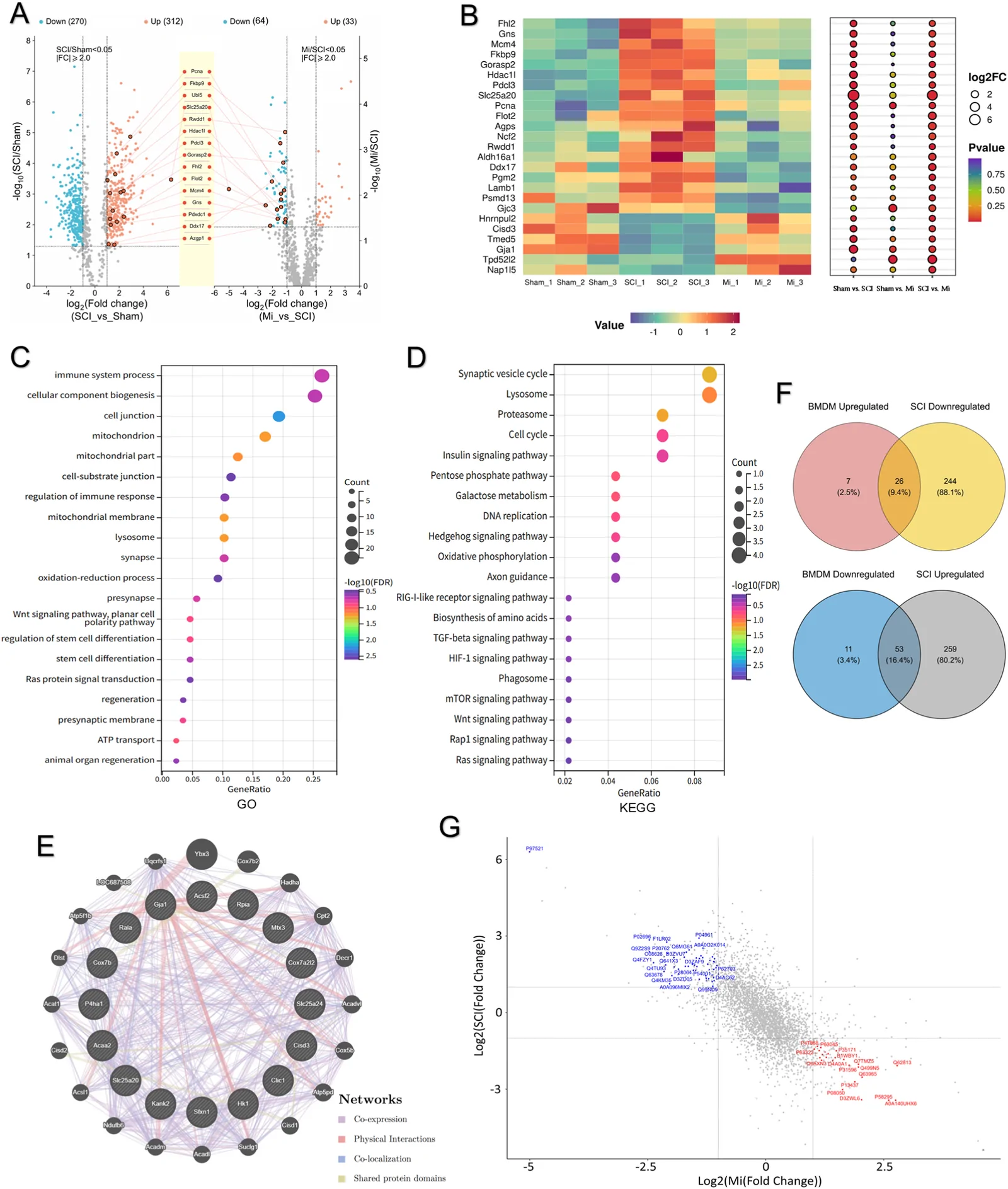
Proteomic analysis in the context of Mi transplantation 3 days after SCI. (**A**) The left plot illustrates DEPs between Sham and SCI samples, highlighting proteins significantly upregulated or downregulated after SCI. The right plot shows DEPs between Mi-treated and SCI samples. A subset of SCI-upregulated proteins overlapped with those downregulated upon Mi treatment, indicating a potential therapeutic reversal effect. The y-axis represents –log_10_(*P*-value), a one-way ANOVA followed by Tukey's test was used to assess statistical significance, n = 3/group. (**B**) Representative heatmap showing protein expression profiles across the Sham, SCI, and Mi treatment groups. (**C**) Gene Ontology (GO) analysis of DEPs, displaying a subset of enriched GO terms, was selected based on biological relevance. (**D**) KEGG pathway analysis of DEPs, identifying key signalling pathways modulated by SCI and Mi treatment. (**E**) Protein–protein interaction (PPI) network generated using GeneMANIA, illustrating mitochondria-related proteins rescued by Mi treatment identified in the analysis. (**F**) Venn diagrams showing overlaps between “SCI-Upregulated” DEPs (proteins upregulated in the SCI group relative to Sham) and “BMDM-Downregulated” DEPs (proteins downregulated in the Mi group relative to SCI), as well as between “SCI-Downregulated” DEPs and “BMDM-Upregulated” DEPs. (**G**) Two-dimensional scatter plot depicting log_2_ fold changes of all protein expression in the SCI group versus Sham (y-axis) and in the Mi group versus SCI (x-axis), demonstrating that Mi treatment partially reversed SCI-induced proteomic alterations.

To explore molecular changes associated with Mi transplantation, we performed proteomic analysis of spinal cord tissues collected three days after SCI. After pre-filtering the features using one-way ANOVA (adjusted *P* < 0.05), globally variable proteins were identified. A dual volcano plot was generated to visualise differentially expressed proteins (Fig. 10A) [22]. The left panel shows proteins altered after SCI compared with the sham condition, whereas the right panel shows DEPs between the Mi-treated and vehicle-treated SCI groups. Notably, a subset of proteins that were upregulated after SCI overlapped with those downregulated following Mi treatment. The heatmap in Fig. 10B further shows distinct protein expression patterns among the sham, vehicle-treated SCI, and Mi-treated SCI groups.

DEPs between the SCI and sham groups were significantly enriched in pathways related to ribosome function, protein processing in the endoplasmic reticulum, tight junction, lysosomal activity, and amino acid biosynthesis. In addition, enrichment was observed in pathways associated with vesicle, transport, and various localization processes, indicating extensive alterations in intracellular trafficking and protein homeostasis following SCI (Fig. S4A and B). DEPs between the vehicle-treated SCI and Mi-treated SCI groups were then selected for further analysis. GO analysis indicated enrichment of biological processes related to immune regulation, synaptic function, mitochondrial organisation, stem cell differentiation, and tissue repair (Fig. 10C). KEGG pathway analysis identified pathways related to axon guidance, synaptic function, and signalling cascades, including mTOR, Hedgehog, and Rap1 pathways (Fig. 10D). Compared with the vehicle-treated SCI group, the Mi-treated group showed altered expression of several synapse-related proteins, including Clstn3, Slc6a5, Slc1a2, Ap2a1, Vamp2, and Syngr3 (Fig. S4C). In addition, several mitochondrial-associated proteins, including Aif, Opa1, Rala, Cox7a2, Hk1, Sfxn1, Cisd3, Acaa2, Acsf2, Gja1, and Cox7b, were increased in the Mi-treated group (Fig. S4D). Given the known involvement of mitochondria in energy metabolism, oxidative stress, and apoptosis after SCI, these proteins represent candidate molecules for future mechanistic validation (Fig. 10E) [33,34].

Venn diagram analysis showed that Mi treatment partially reversed the expression of 53 SCI-upregulated proteins and 26 SCI-downregulated proteins (Fig. 10F and G). Several of these reversed proteins were related to mitochondrial function, consistent with the enrichment analysis. Together, these findings suggest that Mi transplantation partially counteracts SCI-associated proteomic alterations, particularly those related to mitochondrial, synaptic, and immune-regulatory processes.

## 4 Discussion

Spinal cord injury (SCI) triggers a complex secondary injury cascade in which neuroinflammation plays a central role in determining tissue preservation and functional recovery [35]. Macrophages and microglia are key regulators of this response, and their activation states can either exacerbate tissue damage or support repair depending on the local microenvironment [36]. Although macrophages have traditionally been classified into pro-inflammatory M1 and anti-inflammatory M2 phenotypes, this binary model does not fully capture their plasticity in vivo. Instead, macrophages can adopt intermediate or hybrid states in response to complex combinations of inflammatory and reparative signals [10,11].

Secondary injury is widely recognized as a key driver of tissue destruction and functional impairment after SCI; therefore, early modulation of the inflammatory microenvironment represents a rational strategy to attenuate secondary damage during the acute-to-early subacute phase. Building on this rationale and the recognized plasticity of macrophages in the SCI microenvironment, our study suggests that transplantation of reparative-biased macrophages induced ex vivo with LPS, IL-4, and TGF-β can modulate the inflammatory milieu of the injured spinal cord and support early functional recovery in a rat model of SCI. These “Mi” macrophages displayed a mixed activation profile, expressing high levels of anti-inflammatory markers, such as Arg1 and IL-10 while retaining the robust phagocytic activity. This hybrid phenotype aligns with recent findings by Ishida et al., who demonstrated that simultaneous stimulation of macrophages with LPS and IL-4 suppresses iNOS and IL-12 expression, thereby fostering an anti-inflammatory yet functionally active state [37].

The therapeutic relevance of macrophage-based modulation has been supported by several preclinical SCI studies. Transplantation of M2-like macrophages, IL-4-polarised microglia, TUDCA-induced macrophages, or genetically modified macrophages has been reported to reduce inflammation, preserve tissue, and improve functional outcomes after SCI [[38], [39], [40]]. Our findings are broadly consistent with these studies and extend them by showing that ex vivo–programmed Mi macrophages are associated with early inflammatory modulation, tissue preservation, reduced apoptosis, and improved locomotor recovery. Importantly, the inducer group was included to distinguish the effects of local LPS/IL-4/TGF-β delivery from those of Mi cell transplantation. Although inducer administration produced partial effects in some outcomes, Mi transplantation showed broader benefits across histological and behavioural assessments. This suggests that the transplanted cells may provide more sustained or localised modulation of the lesion microenvironment than a single bolus of soluble inducers. However, because the inducer and cell transplantation approaches differ in pharmacokinetics, local retention, and duration of bioactivity, the observed differences between the inducer and Mi groups should not be attributed solely to the presence or absence of Mi cells. These findings suggest that Mi macrophages may contribute to tissue preservation through both phagocytic activity and modulation of the local inflammatory environment. This is consistent with the concept of “immune modulation rather than immune suppression,” in which balanced inflammation is considered important for tissue repair [41]. The increased density of CD206^+^/IBA1^+^ lesion-associated phagocytes after Mi transplantation may reflect a bystander effect on endogenous macrophages and/or microglia, although direct cell-tracing studies are required to confirm this possibility. These findings support further investigation of ex vivo macrophage programming as a potential immunomodulatory strategy for SCI [10].

Mi macrophages generated ex vivo exhibited a hybrid activation profile associated with both inflammatory and reparative programmes. We do not claim that this profile exists endogenously after SCI or that Mi represents a completely novel macrophage state. Rather, our findings indicate that combined LPS, IL-4, and TGF-β stimulation generates an ex vivo-programmed reparative-biased macrophage population that can modulate the injury microenvironment and may provide therapeutic benefit. Because direct in vivo comparisons with canonical M1 and M2 macrophages were not performed, these results should not be interpreted as evidence of superiority over established macrophage phenotypes [11,37].

At the molecular level, the proteomic findings provide exploratory information on pathways associated with Mi treatment. Emerging evidence suggests that hybrid macrophage phenotypes can engage in both glycolytic and oxidative phosphorylation pathways, supporting sustained activity and resilience in the hostile SCI microenvironment [42]. In vitro, our proteomic analyses revealed that Mi-mediated modulation of signalling pathways, including the cAMP and Rap1 pathways, mitochondrial functions, adherens junctions, phagocytosis, and myelination, is well-recognized for its roles in cell survival, protection of the blood-brain barrier, cytoskeletal remodelling, and neural repair [43]. Moreover, consistent with the proteomic findings at the cellular level, mass spectrometry analysis of spinal cord tissues, together with subsequent Western blot validation of representative mitochondrial-related proteins, showed partial reversal of SCI-associated changes in mitochondria-related proteins in the Mi-treated group. Given the role of mitochondria in energy metabolism, oxidative stress, and cell survival, these findings suggest that mitochondrial-associated pathways may be linked to the tissue-preserving effects of Mi transplantation [33]. Nevertheless, these proteomic findings remain associative. Functional validation will be required before definitive mechanistic conclusions can be drawn. Recent studies indicate that inflammatory tissue injury may involve interconnected programmed cell death pathways, including apoptosis, pyroptosis, and necroptosis, as well as ROS/NLRP3-mediated pyroptosis [44,45]. Thus, future studies should determine whether Mi transplantation affects broader inflammatory cell death programmes beyond Cleaved Caspase-3-associated apoptosis.

The association between histological markers and functional recovery further supports the biological relevance of tissue preservation after Mi transplantation. NeuN^+^/Caspase3^+^ cell density showed the strongest predictive performance for functional recovery in the logistic regression model, whereas MBP intensity showed the strongest association with BBB scores in the linear model. These findings suggest that neuronal apoptosis and myelin integrity are closely related to post-injury functional outcomes. Integrating these complementary histological markers may improve the assessment of recovery after SCI and provide useful candidate biomarkers for future translational studies.

Despite these encouraging findings, our study has several limitations that warrant careful consideration. First, the in vivo follow-up period was limited to 14 days after SCI. Therefore, the present study focused on the acute and early subacute phases of SCI, whereas the long-term effects of Mi transplantation during chronic SCI remain to be investigated. Second, although the study compared Mi transplantation with vehicle and inducer administration, it did not include direct transplantation controls using conventional M1-or M2-polarised macrophages or other advanced macrophage therapies, such as TUDCA-induced M2 cells [6]. Therefore, the current data do not establish that Mi macrophages are superior to canonical macrophage phenotypes or other macrophage-based therapies. Rather, Mi should be interpreted as a parallel candidate reparative-biased macrophage population that warrants direct comparison with conventional macrophage phenotypes in future studies. Third, no direct cell-tracing experiments were performed. As a result, the fate, survival, migration, and phenotypic stability of transplanted Mi macrophages remain unclear. Fourth, although the use of cortical neurons in co-culture is a common and practical approach for assessing neuronal responses, cortical neurons and spinal motor neurons may differ in their sensitivity to inflammatory stimuli, injury signals, and receptor expression profiles. Therefore, the ex vivo co-culture findings should be extrapolated to the injured spinal cord environment with caution. The use of LPS in the ex vivo induction protocol also raises translational concerns because of its endotoxin activity. Future studies should explore safer, clinically compatible TLR4 agonists, such as monophosphoryl lipid A (MPLA) [37,46]. Finally, the proposed mitochondrial-, synaptic-, and immune-related pathways should be regarded as hypothesis-generating. Future studies should address these limitations by incorporating longer observation periods, broader phenotypic validation, direct M1 and M2 macrophage transplantation controls, cell-tracing strategies, and mechanistic validation of candidate pathways using pathway inhibition and gain- or loss-of-function approaches. In addition, optimisation of Mi induction conditions, assessment of residual inducers, pharmacokinetic and dose-response evaluation of soluble inducers, and development of good manufacturing practice-compatible protocols will be essential for translational development. Comparative studies using human monocyte-derived or stem cell-derived macrophages may help determine whether a Mi-like reparative-biased phenotype can be generated safely and reproducibly in human cells. Biomaterial carriers or hydrogels may also improve local cell retention and survival within the injured spinal cord.

From a translational perspective, Mi macrophage transplantation may provide an early immunomodulatory strategy for acute SCI, where current treatments remain largely supportive [3,38]. However, the differences between rodent and human SCI microenvironments should be carefully considered. Clinical translation will require several key steps, including the development of GMP-compatible protocols for generating Mi-like macrophages from human monocytes or stem cell-derived precursors. Because LPS raises regulatory and safety concerns, safer TLR4 agonists such as MPLA should be explored for human application [46]. Future studies should also determine whether Mi therapy is more feasible as an autologous or allogeneic product, and whether biomaterial carriers or hydrogels can improve cell delivery, retention, and function within the injured spinal cord [43].

Another important translational challenge is the difference between rodent and human SCI microenvironments. Human SCI involves more complex immune responses, and cross-species differences in monocyte/macrophage subsets and signalling pathways may influence therapeutic efficacy [47]. Therefore, future studies should identify conserved biomarkers of Mi activity and therapeutic response. Integrated proteomic and transcriptomic profiling of rodent and human macrophages, together with CSF or blood analyses from SCI patients, may help define biomarker panels for patient stratification, treatment monitoring, and early-phase clinical study design. Overall, these findings support the potential of Mi macrophage transplantation as an early immunomodulatory approach for experimental SCI. However, further studies are needed to define the long-term fate of transplanted cells, their interaction with host immune populations, and their durability and safety in chronic stages of injury.

## 5 Conclusion

This study evaluated LPS-, IL-4-, and TGF-β-induced reparative-biased macrophages (Mi) as a potential cell therapy for SCI. Mi macrophages exhibited anti-inflammatory, pro-repair, and phagocytic features in vitro, while Mi transplantation was associated with early inflammatory modulation, tissue preservation, reduced apoptosis, and functional recovery after SCI. Proteomic analyses identified candidate mitochondrial-, synaptic-, and immune-related pathways for future validation. Further studies are required to assess long-term outcomes, compare Mi with conventional M1/M2 macrophage transplantation, track transplanted cells, and validate underlying mechanisms.

## Author contribution

AY and XZ conceived the project. XZ, GZ and HW performed experiments. XZ, RH and ML contributed to data analysis. TS and MH provided materials and methods. SK and AU supervised the project. XZ and AY wrote the manuscript, and all other authors edited it. All authors approved the final version of the manuscript.

## Ethical approval

All surgeries and outcome assessments were blinded, and all experimental protocols were approved by the Animal Care Committee of the federal government of Baden Württemberg, Germany (ethic approval code G-285/19).

## Availability of data and materials

The datasets used and/or analysed during the current study are available from the corresponding author on reasonable request. The mass spectrometry proteomics data have been deposited to the ProteomeXchange Consortium via the PRIDE partner repository with the dataset identifier PXD066320. Reviewer access details: project accession: PXD066320 token: R95BXs1j9GJV.

## Declaration of generative AI and AI-assisted technologies in the manuscript preparation process

Generative artificial intelligence (ChatGPT, OpenAI, USA) was used solely for English language editing during manuscript revision, and all authors take full responsibility for the content and conclusions.

## Sources of funding

No external funding was received for this study.

## Declarations of interest

The authors have no conflicts of interest relevant to this article.

## Figures and Tables

**figs1:**
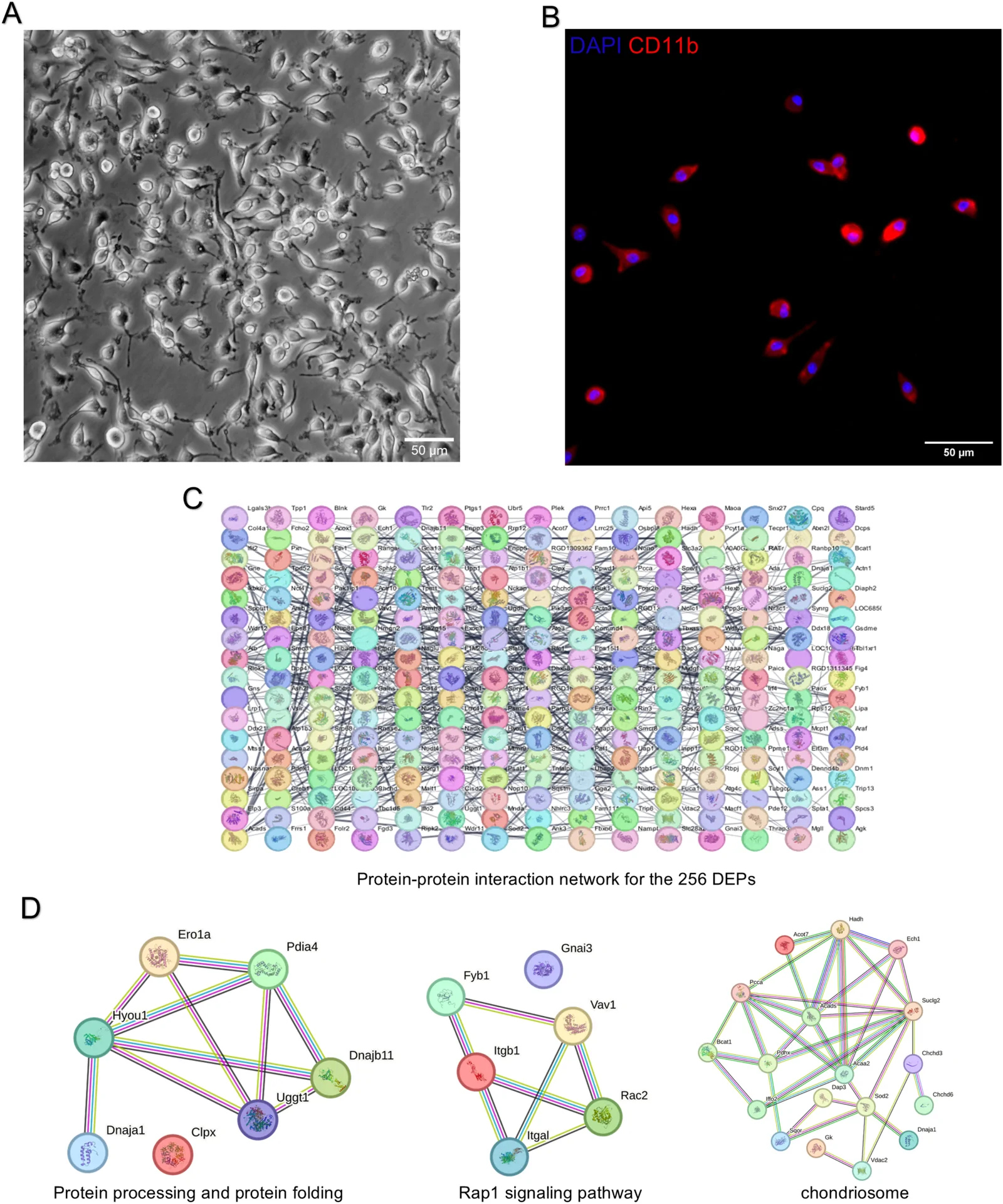


**figs2:**
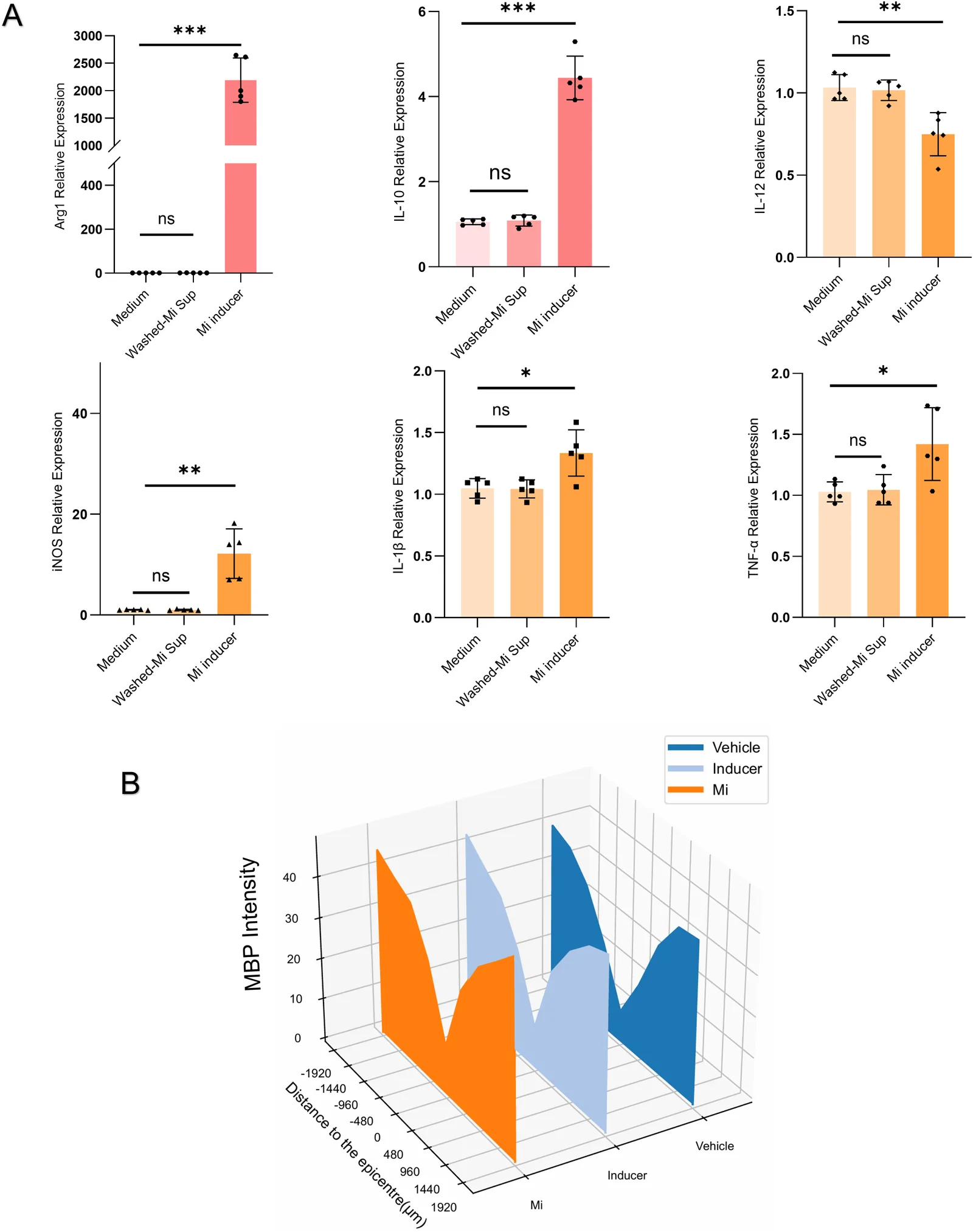


**figs3:**
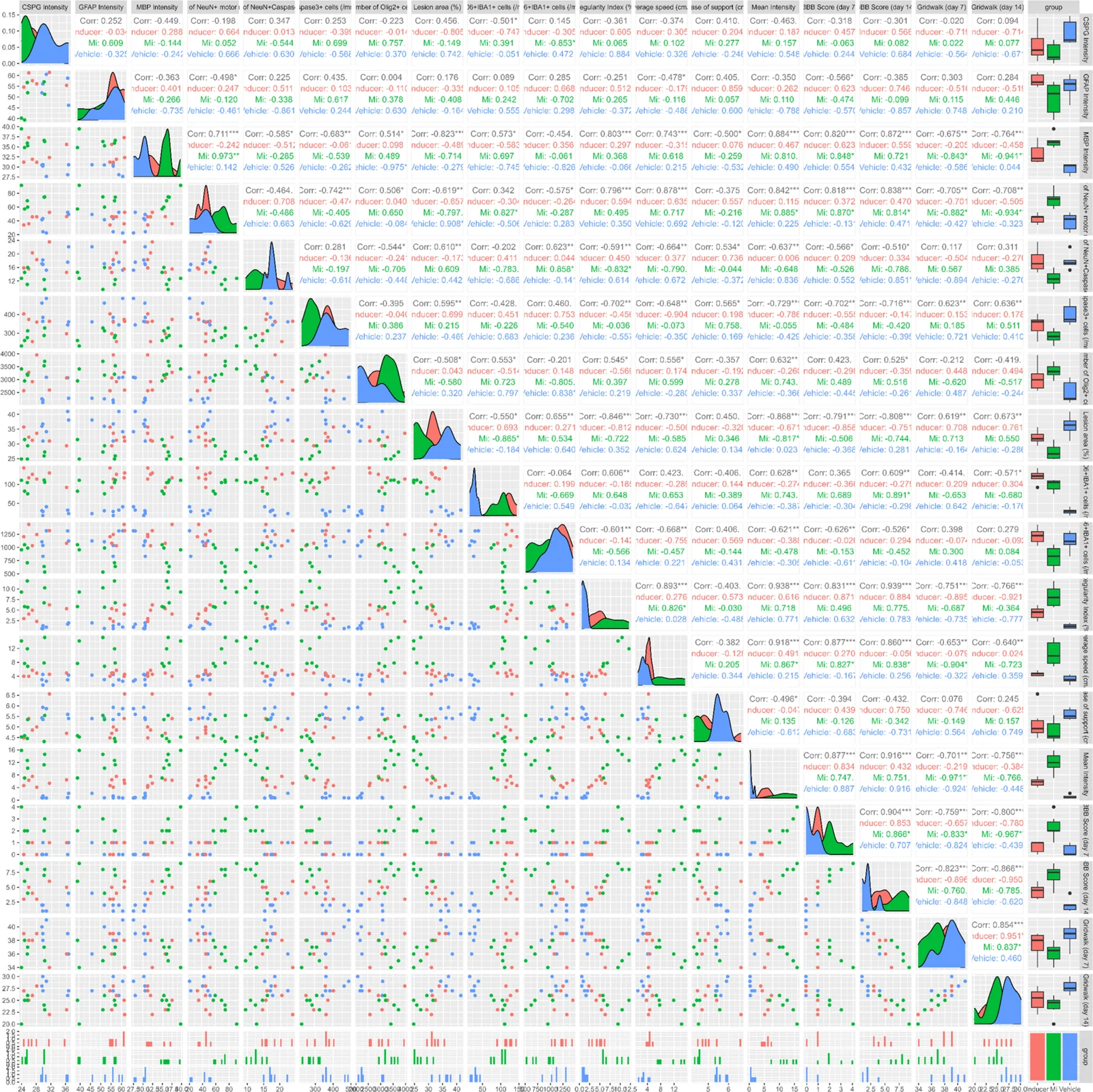


**figs4:**