# Supplementary material for: Transplantation of LPS, IL-4, and TGF-β-induced reparative-biased macrophages promotes early motor functional recovery after spinal cord injury in rats
Source: J Orthop Translat. 2026 Sep 5;60:101219. doi: 10.1016/j.jot.2026.101219 (PMC13572017; doi:10.1016/j.jot.2026.101219)
Supplement: Supplementary file 1 [file mmc1.docx]

## Model metrics

Summary of Model Metrics (rounded to 3 decimals)

| Variable | LM_adjR2 | GLM_AIC | GLM_BIC | GLM_p.val | ROC_AUC | statistic | p.value | Significant |
| --- | --- | --- | --- | --- | --- | --- | --- | --- |
| CSPG Intensity | 0.034 | 18.202 | 19.983 | 0.235 | 0.733 | 1.076 | 0.282 | FALSE |
| GFAP Intensity | 0.095 | 18.468 | 20.248 | 0.191 | 0.733 | 1.223 | 0.221 | FALSE |
| MBP Intensity | 0.745 | 12.806 | 14.586 | 0.143 | 0.911 | 0.810 | 0.418 | FALSE |
| Number of NeuN+ motor neurons | 0.684 | 9.212 | 10.993 | 0.119 | 0.978 | 0.000 | 1.000 | FALSE |
| Number of NeuN+Caspase3+ cells | 0.214 | 7.679 | 9.460 | 0.215 | 0.978 | 0.000 | 1.000 | FALSE |
| Caspase3+ cells (/mm2) | 0.482 | 16.121 | 17.902 | 0.109 | 0.844 | 1.012 | 0.311 | FALSE |
| Number of Olig2+ cells | 0.230 | 16.743 | 18.524 | 0.126 | 0.867 | 1.336 | 0.181 | FALSE |
| CD206+IBA1+ cells (/mm2) | 0.331 | 18.778 | 20.558 | 0.295 | 0.600 | 2.966 | 0.003 | TRUE |
| CD86+IBA1+ cells (/mm2) | 0.231 | 13.670 | 15.451 | 0.049 | 0.933 | 1.183 | 0.237 | FALSE |
