# Supplementary material for: Transplantation of LPS, IL-4, and TGF-β-induced reparative-biased macrophages promotes early motor functional recovery after spinal cord injury in rats
Source: J Orthop Translat. 2026 Sep 5;60:101219. doi: 10.1016/j.jot.2026.101219 (PMC13572017; doi:10.1016/j.jot.2026.101219)
Supplement: Supplementary file 2 [file mmc2.docx]

| Univariate Linear Models | | | | | | | | | |
| --- | --- | --- | --- | --- | --- | --- | --- | --- | --- |
|  | **LM – CSPG** | **LM – GFAP** | **LM – MBP** | **LM – NeuN Motor** | **LM – NeuN+Casp3 Cells** | **LM – Casp3 Cells** | **LM – Olig2 Cells** | **LM – CD206 IBA1 Cells** | **LM – CD86 IBA1 Cells** |
| + p < 0.1, * p < 0.05, ** p < 0.01, *** p < 0.001 | | | | | | | | | |
| (Intercept) | 9.929* | 12.438* | −16.665*** | −1.477 | 10.101*** | 13.286*** | −2.749 | 1.496 | 9.970*** |
|  | (4.378) | (4.825) | (2.979) | (1.017) | (2.443) | (2.198) | (2.962) | (1.073) | (2.292) |
|  | [0.647, 19.211] | [2.209, 22.667] | [−22.980, −10.350] | [−3.633, 0.680] | [4.923, 15.279] | [8.627, 17.945] | [−9.028, 3.530] | [−0.780, 3.771] | [5.111, 14.829] |
| CSPG Intensity | −0.188 |  |  |  |  |  |  |  |  |
|  | (0.149) |  |  |  |  |  |  |  |  |
|  | [−0.503, 0.127] |  |  |  |  |  |  |  |  |
| GFAP Intensity |  | −0.148 |  |  |  |  |  |  |  |
|  |  | (0.089) |  |  |  |  |  |  |  |
|  |  | [−0.336, 0.040] |  |  |  |  |  |  |  |
| MBP Intensity |  |  | 0.638*** |  |  |  |  |  |  |
|  |  |  | (0.090) |  |  |  |  |  |  |
|  |  |  | [0.448, 0.828] |  |  |  |  |  |  |
| Number of NeuN+ motor neurons |  |  |  | 0.116*** |  |  |  |  |  |
|  |  |  |  | (0.019) |  |  |  |  |  |
|  |  |  |  | [0.076, 0.156] |  |  |  |  |  |
| Number of NeuN+Caspase3+ cells |  |  |  |  | −0.349* |  |  |  |  |
|  |  |  |  |  | (0.147) |  |  |  |  |
|  |  |  |  |  | [−0.662, −0.037] |  |  |  |  |
| Caspase3+ cells (/mm2) |  |  |  |  |  | −0.026*** |  |  |  |
|  |  |  |  |  |  | (0.006) |  |  |  |
|  |  |  |  |  |  | [−0.039, −0.013] |  |  |  |
| Number of Olig2+ cells |  |  |  |  |  |  | 0.002* |  |  |
|  |  |  |  |  |  |  | (0.001) |  |  |
|  |  |  |  |  |  |  | [0.000, 0.005] |  |  |
| CD206+IBA1+ cells (/mm2) |  |  |  |  |  |  |  | 0.036** |  |
|  |  |  |  |  |  |  |  | (0.012) |  |
|  |  |  |  |  |  |  |  | [0.011, 0.060] |  |
| CD86+IBA1+ cells (/mm2) |  |  |  |  |  |  |  |  | −0.005* |
|  |  |  |  |  |  |  |  |  | (0.002) |
|  |  |  |  |  |  |  |  |  | [−0.010, −0.001] |
| Num.Obs. | 18 | 18 | 18 | 18 | 18 | 18 | 18 | 18 | 18 |
| R2 | 0.091 | 0.148 | 0.760 | 0.703 | 0.260 | 0.512 | 0.275 | 0.370 | 0.277 |
| R2 Adj. | 0.034 | 0.095 | 0.745 | 0.684 | 0.214 | 0.482 | 0.230 | 0.331 | 0.231 |
| AIC | 87.0 | 85.8 | 63.0 | 66.9 | 83.3 | 75.8 | 82.9 | 80.4 | 82.9 |
| BIC | 89.7 | 88.5 | 65.7 | 69.6 | 86.0 | 78.5 | 85.6 | 83.1 | 85.6 |
| Log.Lik. | −40.509 | −39.923 | −28.515 | −30.447 | −38.658 | −34.905 | −38.467 | −37.202 | −38.452 |
| F | 1.597 | 2.781 | 50.715 | 37.824 | 5.616 | 16.798 | 6.080 | 9.411 | 6.116 |
| RMSE | 2.30 | 2.22 | 1.18 | 1.31 | 2.07 | 1.68 | 2.05 | 1.91 | 2.05 |
