# Supplementary material for: Transplantation of LPS, IL-4, and TGF-β-induced reparative-biased macrophages promotes early motor functional recovery after spinal cord injury in rats
Source: J Orthop Translat. 2026 Sep 5;60:101219. doi: 10.1016/j.jot.2026.101219 (PMC13572017; doi:10.1016/j.jot.2026.101219)
Supplement: Supplementary file 3 [file mmc3.docx]

| Univariate Logistic Models (ORs) | | | | | | | | | |
| --- | --- | --- | --- | --- | --- | --- | --- | --- | --- |
|  | **GLM – CSPG** | **GLM – GFAP** | **GLM – MBP** | **GLM – NeuN Motor** | **GLM – NeuN+Casp3 Cells** | **GLM – Casp3 Cells** | **GLM – Olig2 Cells** | **GLM – CD206 IBA1 Cells** | **GLM – CD86 IBA1 Cells** |
| + p < 0.1, * p < 0.05, ** p < 0.01, *** p < 0.001 | | | | | | | | | |
| (Intercept) | 951.291 | 123.764 | 0.000 | 0.000 | 51031393684.069 | 781.042 | 0.000+ | 0.026 | 350.762+ |
|  | (6574.500) | (597.886) | (0.000) | (0.000) | (1023291861940.849) | (3799.027) | (0.000) | (0.058) | (1248.306) |
| CSPG Intensity | 0.738 |  |  |  |  |  |  |  |  |
|  | (0.189) |  |  |  |  |  |  |  |  |
| GFAP Intensity |  | 0.885 |  |  |  |  |  |  |  |
|  |  | (0.083) |  |  |  |  |  |  |  |
| MBP Intensity |  |  | 2.579 |  |  |  |  |  |  |
|  |  |  | (1.669) |  |  |  |  |  |  |
| Number of NeuN+ motor neurons |  |  |  | 1.267 |  |  |  |  |  |
|  |  |  |  | (0.192) |  |  |  |  |  |
| Number of NeuN+Caspase3+ cells |  |  |  |  | 0.141 |  |  |  |  |
|  |  |  |  |  | (0.223) |  |  |  |  |
| Caspase3+ cells (/mm2) |  |  |  |  |  | 0.974 |  |  |  |
|  |  |  |  |  |  | (0.016) |  |  |  |
| Number of Olig2+ cells |  |  |  |  |  |  | 1.003 |  |  |
|  |  |  |  |  |  |  | (0.002) |  |  |
| CD206+IBA1+ cells (/mm2) |  |  |  |  |  |  |  | 1.022 |  |
|  |  |  |  |  |  |  |  | (0.021) |  |
| CD86+IBA1+ cells (/mm2) |  |  |  |  |  |  |  |  | 0.992* |
|  |  |  |  |  |  |  |  |  | (0.004) |
| Num.Obs. | 18 | 18 | 18 | 18 | 18 | 18 | 18 | 18 | 18 |
| AIC | 18.2 | 18.5 | 12.8 | 9.2 | 7.7 | 16.1 | 16.7 | 18.8 | 13.7 |
| BIC | 20.0 | 20.2 | 14.6 | 11.0 | 9.5 | 17.9 | 18.5 | 20.6 | 15.5 |
| Log.Lik. | −7.101 | −7.234 | −4.403 | −2.606 | −1.840 | −6.061 | −6.372 | −7.389 | −4.835 |
| F | 1.410 | 1.712 | 2.141 | 2.430 | 1.540 | 2.574 | 2.340 | 1.098 | 3.884 |
| RMSE | 0.35 | 0.35 | 0.29 | 0.23 | 0.19 | 0.33 | 0.35 | 0.37 | 0.30 |
